# Toward a Comprehensive Analysis of Posttranscriptional Regulatory Networks: a New Tool for the Identification of Small RNA Regulators of Specific mRNAs

**DOI:** 10.1128/mBio.03608-20

**Published:** 2021-02-23

**Authors:** Kook Han, Stephen Lory

**Affiliations:** a Department of Microbiology, Harvard Medical School, Boston, Massachusetts, USA; Emory University School of Medicine

**Keywords:** discovery of multiple small RNA regulators, *rpoS*, *E. coli*, *P. aeruginosa*, *V. cholerae*, rGRIL-seq, proximity ligation

## Abstract

A number of computational or experimental tools have been developed to identify targets of small RNA (sRNA) regulation. Here, we modified one of these methods, based on *in vivo* proximity ligation of sRNAs bound to their targets, referred to as rGRIL-seq, that can be used to capture sRNA regulators of a gene of interest. Intracellular expression of bacteriophage T4 RNA ligase leads to a covalent linking of sRNAs base-paired with mRNAs, and the chimeras are captured using oligonucleotides complementary to the mRNA, followed by sequencing. This allows the identification of known as well as novel sRNAs. We applied rGRIL-seq toward finding sRNA regulators of expression of the stress response sigma factor RpoS in Escherichia coli, Pseudomonas aeruginosa, and Vibrio cholerae. In E. coli, we confirmed the regulatory role of known sRNAs and discovered a new negative regulator, asYbiE. When applied to P. aeruginosa and V. cholerae, we identified two novel sRNAs (s03661 and s0223) in P. aeruginosa and two known sRNAs (TfoR and Vcr043) in V. cholerae as direct regulators of *rpoS*. The use of rGRIL-seq for defining multiple posttranscriptional regulatory inputs into individual mRNAs represents a step toward a more comprehensive understanding of the workings of bacterial regulatory networks.

## INTRODUCTION

Bacterial small regulatory RNAs (sRNAs) are typically ∼40 to 400 nt in length and act as gene-expression regulators by various mechanisms (1, 2). Extensive studies over the past 3 decades have shown that they modulate a wide range of physiological responses in bacteria. The discovery of the large number of bacterial sRNAs led to an appreciation of the role of posttranscriptional regulation in controlling prokaryotic gene expression, rivaling transcription factors in their abundance and in the number and functional diversity of their regulatory targets.

The basic molecular mechanism responsible for regulating gene expression is based on base-pairing between sRNAs and their mRNA targets, positively or negatively influencing translation and/or transcript stability. sRNAs are typically transcribed as unlinked genes, frequently from intergenic regions but occasionally overlapping coding sequences. However, sRNAs can be also generated from mRNAs by processing or by transcription originating from sites within open reading frames (reviewed in reference 3). In addition to directly regulating the initiation of translation and mRNA stability, certain sRNAs can perform other regulatory functions, including modulating the activity of regulatory proteins (e.g., CsrA/RsmA) or by encoding small peptides (e.g., SgrT) with their own regulatory targets (4–6). The ability of sRNAs to control mRNAs depends on their transcription, which is frequently regulated (7). However, recent work has shown that sRNAs can also interact with other transcripts, including different sRNAs and fragments derived from mRNAs or tRNAs, and this sequestration (“sponging”) mechanism can reverse their regulatory effect on mRNAs (8).

In order to fully describe sRNA regulons, several computational and experimental methods have been developed to define specific regulatory sRNA-mRNA interactions. Most experimental approaches involve capturing complexes between sRNAs and their mRNA targets using antibodies for the RNA binding proteins (Hfq, RNase E, or ProQ; called RIL-seq or CLASH) (9–11) or copurifying them using sRNA fused to the MS2 tag with MS2-affinity purification (called MAPS) (12). Unlike the coimmunoprecipitation or affinity purification-based approaches, we have previously developed the GRIL-seq method, allowing the identification of targets of any single sRNA following its coexpression with T4 RNA ligase in a bacterial cell (13). In GRIL-seq, the chimeras are captured by binding to an oligonucleotide complementary to the sRNA and the ligation products consist of distinct mRNAs or different fragments of the same mRNA. Interactions between two sRNAs or a sponging interaction between an sRNA and other transcript fragments (e.g., derived from tRNA) can be also detected.

While a single sRNA can target multiple mRNAs, it also has been known that a single mRNA can be regulated by more than one sRNA. For example, in Escherichia coli, bacterial motility is negatively regulated by four sRNAs, ArcZ, OmrA, OmrB, and OxyS, and positively by McaS, through a base-pairing interaction with the 5′ untranslated region (UTR) of *flhDC* mRNA, encoding the master regulator of flagellar genes (14). The expression of CsgD, a central regulator of formation of curli and consequently a global regulator of biofilm genes in E. coli, is regulated from a central hub located in the 5′ UTR of the *csgD* transcript, targeted by seven sRNAs (OmrA/B, McaS, RprA, RydC, GcvB, and RybB) (15, 16). The two sRNAs MgrR and RyhB*^Ec^* exert their effects by directly base-pairing with the 5′ region of the *grlRA* operon encoding the transcription factors regulating the expression of the locus of enterocyte effacement in enteropathogenic E. coli (17). One of the best-studied mRNAs regulated by multiple sRNAs is *rpoS* of E. coli, whose transcript is subject to positive or negative regulation by six sRNAs, DsrA, RprA, ArcZ, CyaR, MgrR, and OxyS, and the first four are known to act by direct base-pairing with the 5′ UTR of the *rpoS* mRNA (18–24). Detecting interactions of mRNAs suspected of being targeted by multiple sRNAs of unknown origin or derived by processing of mRNAs is not trivial; it requires modifications of existing experimental or computational methods, and we provide one such approach here.

In this work, we report another variation of the GRIL-seq method, referred to as reverse GRIL-seq (rGRIL-Seq), where we use proximity ligation of base-paired sRNA-mRNA complexes to identify multiple sRNA regulators of mRNAs of interest. Not only does this method provide the means of identifying direct multiple sRNA regulators of a single mRNA, but it can serve as a tool for mining transcriptomes for previously unannotated sRNAs. We applied this approach toward identifying sRNAs controlling the expression of RpoS in three different bacterial species and found diversity in types of sRNAs that control the expression of a highly conserved stationary-phase sigma factor.

## RESULTS

### The rGRIL-seq method.

**FIG 1:**
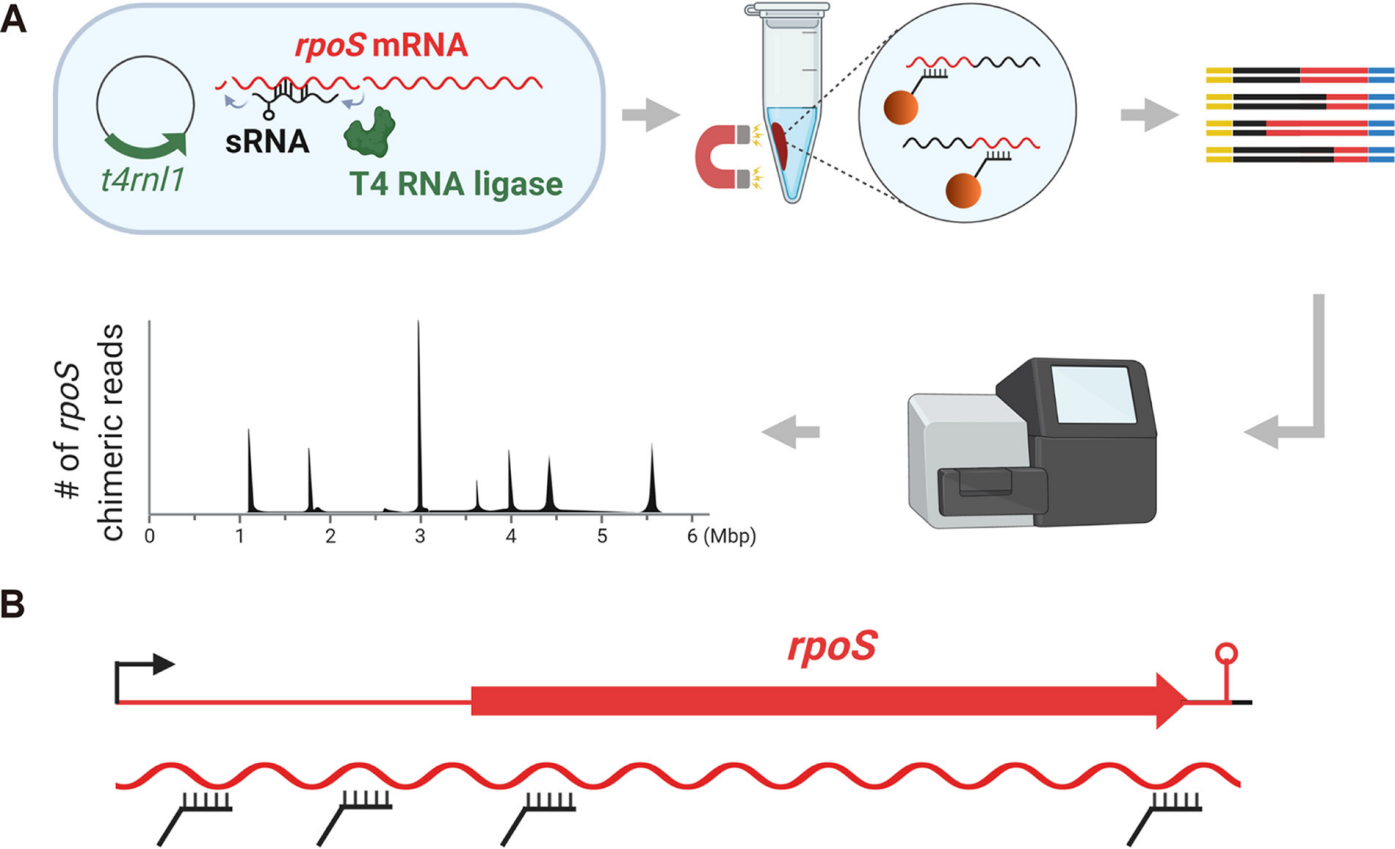
Overview of the rGRIL-seq method to identify sRNAs interacting with *rpoS* mRNA *in vivo*. (A) The sRNA-*rpoS* chimeras are created by base-paired transcripts covalently linked at their 3′ and 5′ ends by the action of T4 RNA ligase (indicated by a curved arrow) in bacteria expressing this enzyme from a plasmid carrying the *t4rnl1* gene. The *rpoS* chimeric RNAs are enriched with magnetic beads bound to multiple mRNA complementary oligonucleotides. The RNA library is constructed from the enriched chimeric RNAs and subjected to Illumina paired-end sequencing. The chimeric reads are mapped and linked to specific genomic locations other than the *rpoS* gene and are represented as peaks on the genome mapping. (B) The multiple complementary oligonucleotides were designed for annealing to the *rpoS* in chimeric RNAs at 4 different locations.

In the previous study, we developed a method for the identification of direct targets of sRNAs in living cells (GRIL-seq), where an sRNA is ectopically expressed in bacteria coexpressing T4 RNA ligase, and the enrichment of the chimeric RNAs (sRNA-target RNA ligation products) was accomplished using a bead-immobilized single oligonucleotide complementary to the specific sRNA (13). In order to identify sRNA regulators targeting a specific mRNA, we altered the GRIL-seq protocol at the enrichment step by reversing the chimera capture strategy; we refer to this modified method as reverse GRIL-seq (rGRIL-seq) (Fig. 1). For the rGRIL-seq approach, we designed multiple oligonucleotides complementary to the different regions of an mRNA sequence and utilize these for bead capture and enrichment of mRNA fragments that are covalently linked to their sRNA regulators by the action of T4 RNA ligase (Fig. 1A). We reasoned that in the absence of any information on ribosome occupancy or the degradation patterns of a specific mRNA, spacing of the segments complementary to the capture oligonucleotides should be random, with a preference given to the 5′ region of mRNAs including the 5′ UTR (Fig. 1B).

**FIG 2:**
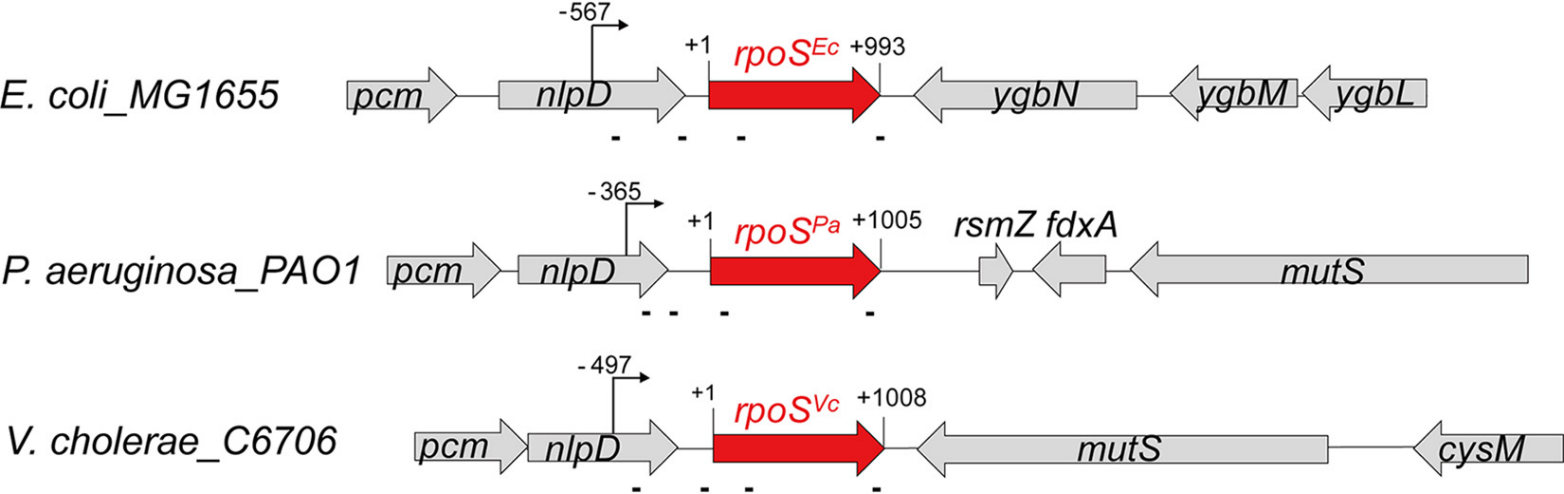
The *rpoS* locus in E. coli, P. aeruginosa, and V. cholerae. The transcription start site of the *rpoS* (red) gene in the *nlpD* gene with arrows was determined by EcoCyc (for E. coli MG1655) and our unpublished RNA-seq data (for P. aeruginosa PAO1 and V. cholerae C6706). The four lines below each *rpoS* gene show the approximate locations of the binding sites for the multiple complementary oligonucleotides used for the enrichment of chimeras.

To test whether rGRIL-seq is a reliable method for identifying sRNA regulators of specific mRNAs, we chose to study the regulation of the *rpoS* transcript in three bacterial species: E. coli, Vibrio cholerae, and Pseudomonas aeruginosa. The stress-responsive sigma factor RpoS has a similar regulatory function in all *Proteobacteria*. In E. coli, several sRNAs controlling the expression of *rpoS* translation and stability have been identified and extensively studied; this allowed for validation of the rGRIL-seq with known as well as previously uncharacterized sRNAs. The E. coli *rpoS* gene is found adjacent to the *nlpD* gene, encoding an outer membrane lipoprotein (Fig. 2). The coding sequence of NlpD contains the major *rpoS* promoter located 567 bp from the start codon for RpoS. This overall genetic organization is conserved among many Gram-negative microorganisms, including P. aeruginosa and V. cholerae, where the transcription start site and the unusually long 5′ UTR are also located within the *nlpD* gene. At the amino acid level, RpoS is conserved (67 to 74% [see Fig. S1A and B in the supplemental material]) as expected from proteins which carry out the same function. However, the nucleotide sequences of the 5′ UTR (55 to 62%), the open reading frame (ORF, 62 to 68%), and 3′ UTR (60 to 63%) are less conserved (Fig. S1C to H). The low conservation of the regulatory sequences, particularly in the 5′ UTR that serves as potential target sequence for base-paring regulatory sRNAs, suggests that they may be recognized by different sRNAs in each species. Not surprisingly, homologs of the known sRNA regulators of E. coli *rpoS* cannot be identified in *Vibrio* or *Pseudomonas* species.

10.1128/mBio.03608-20.2 FIG S1 Comparison of RpoS protein sequences and *rpoS* mRNA sequences between three bacterial species (E. coli MG1655, P. aeruginosa PAO1, and V. cholerae C6706). (A) Protein sequence alignment. (B) Percent identity matrix (up) and guide tree (down) of protein. (C to E) RNA sequence alignment of 5′ UTR (C), ORF (D), and 3′ UTR (E). (F to H) Guide tree (up) and percent identity matrix (down) of 5′ UTR (F), ORF (G), and 3′ UTR (H) comparisons. All were generated by Clustal Omega (https://www.ebi.ac.uk/Tools/msa/clustalo/). Download FIG S1, PDF file, 0.5 MB. Copyright © 2021 Han and Lory. 2021 Han and Lory. https://creativecommons.org/licenses/by/4.0/ This content is distributed under the terms of the Creative Commons Attribution 4.0 International license.

We carried out the rGRIL-seq procedure as described in detail in Materials and Methods. Briefly, for each *rpoS* transcript, we designed 4 oligonucleotides complementary to sequences in the *rpoS* mRNA to be used for the enrichment of the *rpoS*-sRNA chimeras, with their approximate location indicated in Fig. 1B and Fig. 2. We isolated total RNAs from the cells ectopically expressing T4 RNA ligase in actively dividing cells (optical density at 600 nm [OD_600_] of ∼0.4), referred to as exponential-phase cultures (Ex.) and high-density cells (OD_600_ of ∼3.5), referred to as stationary-phase cultures (St.). The *rpoS* chimeric RNAs were enriched from the total RNAs using the four oligonucleotides and were captured on magnetic beads. The cDNAs of these chimeras were sequenced and analyzed by mapping them to the genomes of each bacterial species.

### sRNAs regulating E. coli *rpoS*.

We first carried out the rGRIL-seq in E. coli by extracting RNA from three independent cultures, each grown to exponential (Ex.) and early stationary (St.) phases. We obtained an average of 9,264 and 14,052 *rpoS*-chimeric reads for logarithmic and stationary phases, respectively (Table S1A).

10.1128/mBio.03608-20.9 TABLE S1 (A) Chimeric *rpoS* mRNAs obtained from cells expressing endogenous *rpoS.* Total reads refer to total number of paired-end sequencing reads obtained from the Illumina DNA library constructed with RNAs after enrichment of *rpoS* chimeras. The *rpoS* mapped reads refer to all reads including nonchimeric *rpoS* and *rpoS* chimeras which are mapped to the *rpoS* gene corresponding to *rpoS* transcriptional units including 5′ and 3′ untranslated regions (UTRs). The *rpoS* chimeric reads are obtained by mapping all the *rpoS* mapped reads to the reference genome lacking the *rpoS* transcription unit (see details in Text S1). The enrichment efficiency is the *rpoS* mapped reads, divided by the total reads, and expressed as a percentage. The percentage of the chimeric RNAs is calculated by dividing the *rpoS* chimeric reads by *rpoS* mapped reads including nonchimeric *rpoS* and *rpoS* chimeras. (B) RNAs interacting with *rpoS* mRNA in E. coli, P. aeruginosa, and V. cholerae identified by rGRIL-seq. Max coverage stands for the height of peak created by mapping chimeric reads. Table includes all RNAs observed in more than max coverage 35 (average) in E. coli and V. cholerae and max coverage 2 (average) in P. aeruginosa. Download Table S1, PDF file, 0.3 MB. Copyright © 2021 Han and Lory. 2021 Han and Lory. https://creativecommons.org/licenses/by/4.0/ This content is distributed under the terms of the Creative Commons Attribution 4.0 International license.

**FIG 3:**
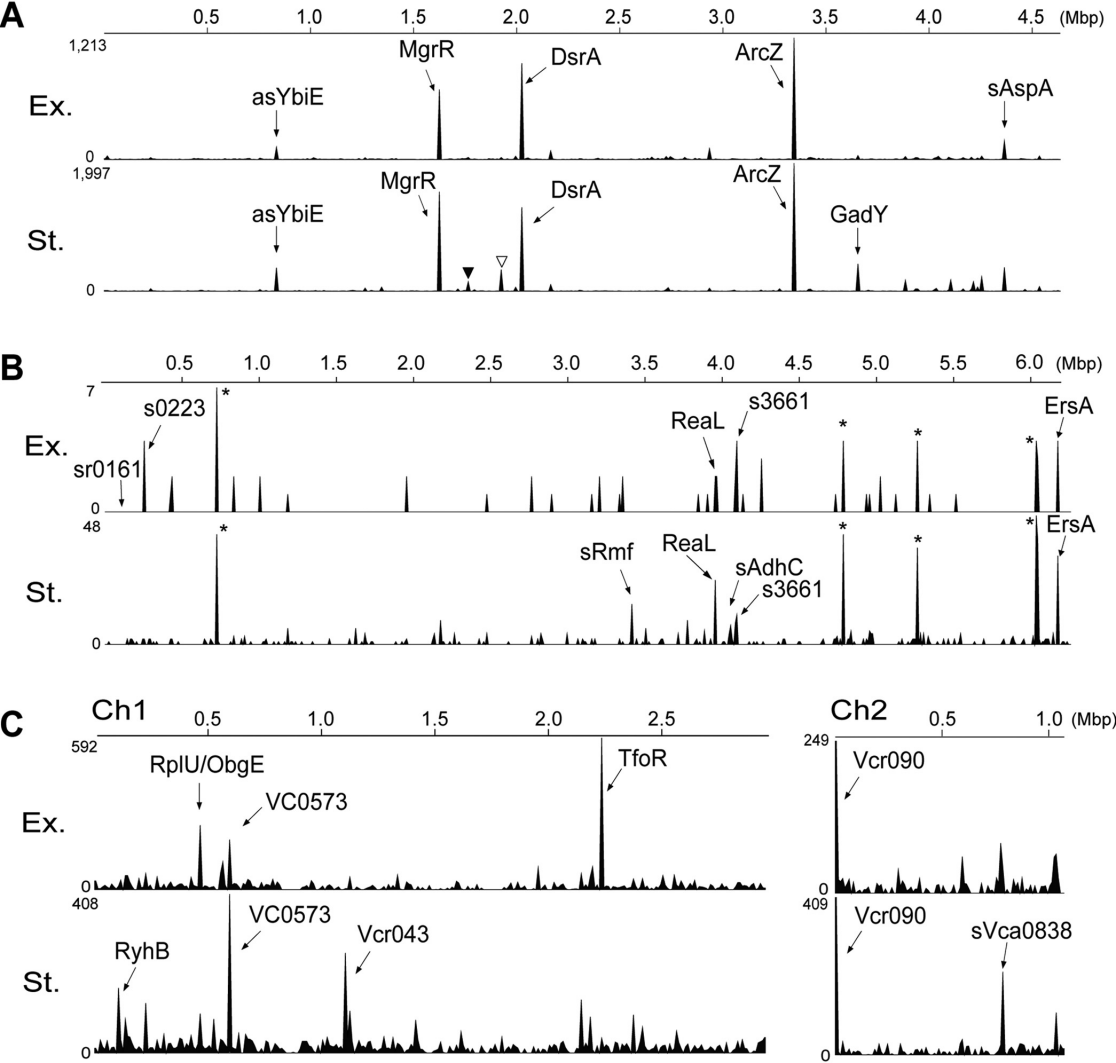
Identification of sRNAs interacting with *rpoS* mRNA in E. coli, P. aeruginosa, and V. cholerae by rGRIL-seq. The sRNAs are mapped on the respective chromosomes at the location of the corresponding genes. Arrows point to the top 5 RNAs ligated to *rpoS* in exponential (Ex.) and stationary (St.) phase in E. coli (A), P. aeruginosa (B), and V. cholerae (C). An inverted triangle in black and white shown in panel A indicates the mapping of RprA-rpoS and SdsR-rpoS, respectively. The asterisks shown in panel B indicate peaks corresponding to 23S and 16S rRNAs.

**FIG 4:**
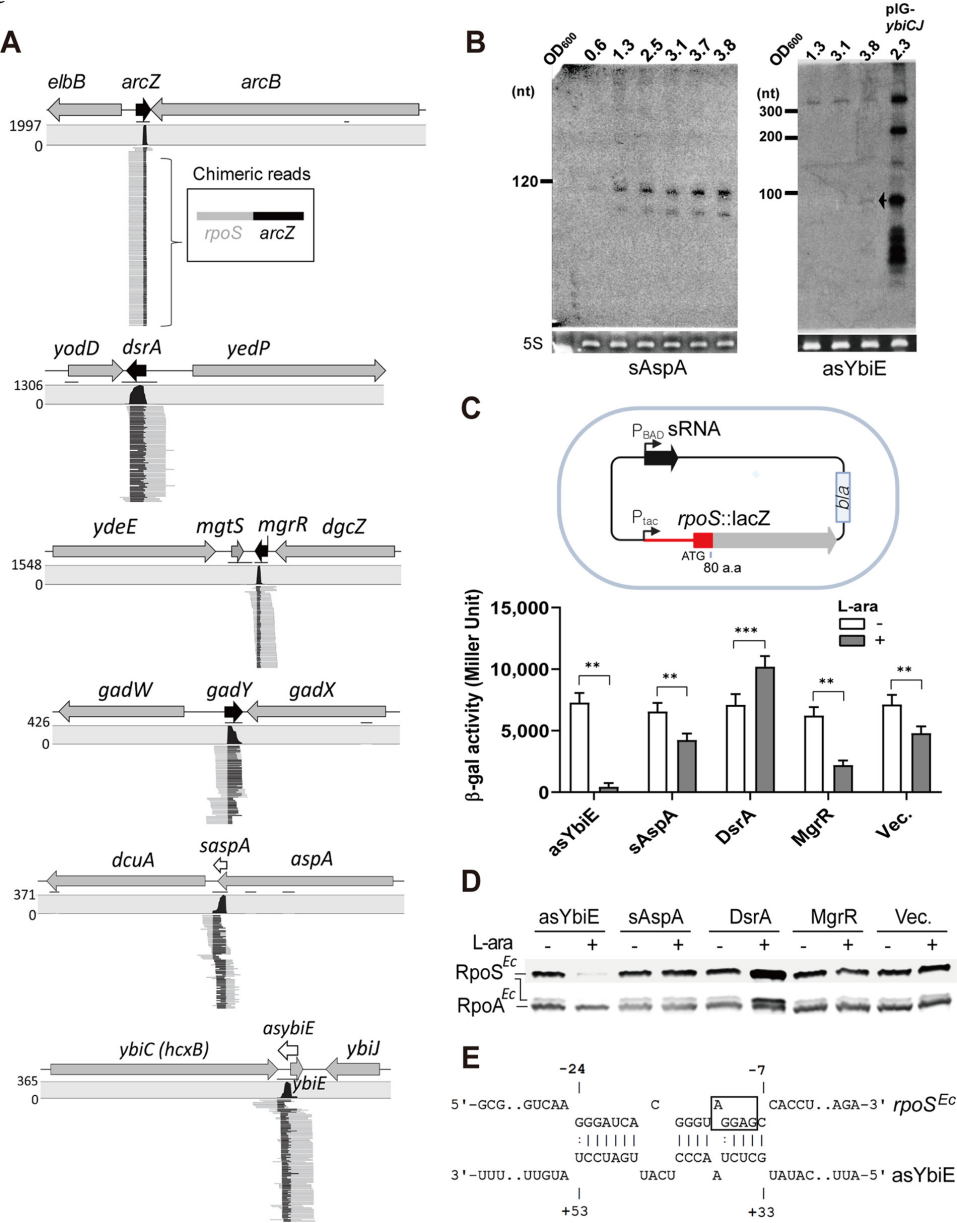
rGRIL-seq in E. coli identifies small RNAs interacting with *rpoS* mRNA. (A) The genomic locations of 6 different RNAs identified as chimeras with *rpoS* by rGRIL-seq, ranked with the top 5 in exponential and stationary phase. An example of a single *rpoS* chimeric read containing ArcZ is shown in a box with *rpoS* (gray) and *arcZ* (black) sequences. The sRNAs annotated in previous studies are represented as black arrows while the sRNAs annotated in this study are represented as white arrows. (B) Northern blot analysis of two *rpoS*-interacting small transcripts (sAspA and asYbiE) at different growth stages in LB medium. Total RNAs were obtained from either the E. coli wild-type strain (MG1655) or the wild type carrying the multicopy plasmid cloned with the intergenic region of *ybiC* or *ybiJ*. The small size of asYbiE (∼100 nt) with a weak signal is marked with an arrow. 5S rRNAs stained with SYBR green were used as a loading control. (C) The β-galactosidase activity monitored when each sRNA is overexpressed (gray) or not (white) in E. coli carrying the plasmid pKH24, which contains both the IPTG-inducible *rpoS^Ec^*::*lacZ* reporter gene and the l-arabinose-inducible sRNA gene. The plasmid (Vec.) lacking sRNA was used as a negative control. Data are shown as mean ± SD for three biological replicates. Statistical comparisons were performed using Student’s *t* test. **, *P* ≤ 0.01; ***, *P* ≤ 0.001. (D) Western blot analysis of endogenous RpoS*^Ec^* levels detected by anti-RpoS*^Ec^* antibodies, using the total proteins isolated from the same cultures used for the β-galactosidase assay. RpoA*^Ec^* was used as a loading control. (E) Base-pairing between asYbiE and *rpoS* mRNA. The Shine-Dalgarno sequence of *rpoS* mRNA is boxed, and the numbers (−24 and −7) over the sequence of *rpoS* mRNA are the nucleotide distances relative to the translational start site.

Mapping the chimeric reads on the genome of E. coli MG1655 allowed us to identify several transcripts preferentially ligated to the *rpoS* mRNA (Fig. 3A). The enrichment for several RNAs was detected in both growth phases, while certain sRNA transcripts ligated to the *rpoS* mRNA were isolated in either the exponential or stationary phase. Table 1 shows the list of top 5 sRNAs ranked by number of reads from sequencing of the *rpoS*-containing chimeras. We identified a total of 6 different *rpoS* chimeric RNAs in the top 5 chimeric reads in each growth phase (Table 1A and Fig. 4A). These include the two known sRNA regulators of *rpoS* (DsrA and ArcZ [19, 21]), while the other two well-characterized sRNAs, RprA and CyaR (18, 19, 23, 24), were also identified but in lesser abundance and ranked 12th and 14th in the stationary phase, respectively (Table S1B). Previously, Moon and Gottesman identified additional regulators of *rpoS* following overexpression of a set of sRNAs and determined their effect on an *rpoS^Ec^-lacZ* translational fusion (24). Among these sRNAs, MgrR and GadY were also found in the *rpoS* chimeras, demonstrating that their effect on the *rpoS^Ec^-lacZ* expression was the consequence of a direct base-pairing interaction between the sRNA and the *rpoS* 5′ UTR. Interestingly, they also showed that the strongest repressive effect on the *rpoS^Ec^-lacZ* fusion was exerted by overexpression of OxyS; yet, the *rpoS* chimera with this sRNA was detected in only a very small number of reads in rGRIL-seq (7 and 3 chimeras of OxyS in Ex. and St., respectively), presumably supporting the indirect regulatory mechanism of this sRNA (25).

**TABLE 1:** RNAs interacting with *rpoS* mRNA in E. coli (A), P. aeruginosa (B), and V. cholerae (C) identified by rGRIL-seq

A. rpoS*^Ec^*						
Rank	Name	Max coverage^*a*^	Mapping location of chimeras	Flanking genes		Reference
**Ex.**				5′	3′	
1	ArcZ	988	sRNA	*elbB*	*arcB*	20
2	DsrA	780	sRNA	*yodD*	*yedP*	21
3	MgrR	606	sRNA	*mgtS*	*dgcZ*	53
4	sAspA	126	3′ RNA of *aspA* mRNA	*dcuA*	*fxsA*	
5	asYbiE	111	Antisense RNA of *ybiE* mRNA	*ybiC*	*ybiJ*	
**St.**				5′	3′	
1	ArcZ	1,601	sRNA	*elbB*	*arcB*	20
2	MgrR	1,115	sRNA	*mgtS*	*dgcZ*	53
3	DsrA	916	sRNA	*yodD*	*yedP*	21
4	GadY	369	sRNA	*gadW*	*gadX*	54
5	asYbiE	315	Antisense RNA of *ybiE* mRNA	*ybiC*	*ybiJ*	

B. rpoS*^Pa^*						
Rank	Name	Max coverage^*a*^	Mapping location of chimeras	Flanking genes		Reference
**Ex.**				5′	3′	
1	ErsA	12	sRNA	*PA5492*	*PA5493*	37
2	s3661	3	3′ RNA of *PA3661* mRNA	*PA3661*	*PA3662*	
3	ReaL	2	sRNA	*PA3535*	*PA3536*	55
4	sr0161	2	sRNA	*PA1060*	*opdP*	38
5	s0223	2	3′ RNA of *PA0223* mRNA	*PA0222*	*PA0224*	
**St.**				5′	3′	
1	ErsA	24	sRNA	*PA5492*	*PA5493*	37
2	ReaL	15	sRNA	*PA3535*	*PA3536*	55
3	sRmf	9	3′ RNA of *rmf* mRNA	*PA3049*	*PA3050*	
4	sAdhC	7	3′ RNA of *adhC* mRNA	*PA3628*	*PA3630*	
5	s3661	6	3′ RNA of *PA3661* mRNA	*PA3661*	*PA3665*	

C. rpoS*^Vc^*						
Rank	Name	Max coverage^*a*^	Mapping location of chimeras	Flanking genes		Reference
**Ex.**				5′	3′	
1	TfoR	564	sRNA	*VC2078*	*VC2080*	45
2	Vcr090	200	sRNA	*VCA0002*	*VCA0003*	43
3	RplU	177	5′ RNA of *rplU* mRNA	*VC0434*	*rpmA*	
4	VC0573	134	5′ RNA of *VC0573* mRNA	*rpsL*	*VC0574*	
5	ObgE	100	5′ RNA of *obgE* mRNA	*rpmA*	*VC0438*	
**St.**				5′	3′	
1	Vcr090	276	sRNA	*VC2078*	*VC2079*	43
2	VC0573	222	5′ RNA of *VC0573* mRNA	*rpsL*	*VC0574*	
3	Vcr043	170	sRNA	*VC1045*	*VC1046*	43
4	sVca0838	123	IGR	*VCA0838*	*VCA0840*	
5	RyhB*^Vc^*	98	sRNA	*VC0106*	*VC0108*	56

a Max coverage stands for the height of peak created by mapping chimeric reads.

We also found two transcripts (sAspA and asYbiE) ligated to E. coli *rpoS* mRNA in the rGRIL-seq analysis (Fig. 3A and Table 1A). Both *rpoS* chimeras were detected in exponential as well as stationary phases at similar levels (Fig. 3A). The sAspA corresponds to the 3′ region of the *aspA* gene encoding aspartate ammonia-lyase (Fig. 4A), which carries out the reversible conversion of l-aspartate to fumarate and ammonia under catabolite repression. The *aspA* transcript (∼1,600 nt) terminates at an inverted repeat immediately downstream of the *aspA* gene (26, 27), as shown in Fig. S2A. We speculate that the sAspA is an sRNA derived from the 3′ end of the *aspA* mRNA; such regulatory transcripts have been previously identified in several other bacteria (28–32), and they are orginated either by transcription from an internal promoter of the gene (Type I) or by processing of the parental mRNA (Type II) (33). Our analysis of this gene identified a putative promoter sequence upstream of the stop codon of the *aspA* gene (Fig. S2A); this would make the sAspA a Type I 3′-derived sRNA. Interestingly, unlike other sRNA-*rpoS* chimeric transcripts, mapping of the *rpoS* sequences in the *rpoS*-sAspA ligation products on the *rpoS* gene showed an enrichment of the chimeras at the 3′ end, near the terminator sequence of *rpoS* mRNA (Fig. S2B and Fig. S4). This implies that the sAspA may interact with the 3′ region of *rpoS* mRNA, allowing us to predict the likely base-pairing between them using RNA-RNA interaction algorithm IntaRNA (34). The predicted base-pairing includes the sequence following the stop codon of *rpoS* and part of the 3′ UTR (hybridization energy, −11.14 kcal/mol, Fig. S2C).

10.1128/mBio.03608-20.3 FIG S2 Genomic location of sAspA and interaction with 3′ UTR of *rpoS* mRNA. (A) The genomic locus of the 3′-derived sRNA, sAspA. The putative promoter (−10 and −35) is indicated in gray, and the sAspA sequence is indicated in bold. The stop codon of *aspA* and start codon of *dcuA* are represented as lowercase and underlined. The terminator sequence of sAspA is shown in arrows. (B) The enrichment of sAspA-*rpoS* chimeric reads on 3′ UTR of *rpoS*. The terminator of *rpoS* is indicated with arrows and “T.” (C) The predicted base-pairing of sAspA with *rpoS* mRNA. The stop codon of *rpoS* is underlined. Download FIG S2, PDF file, 0.3 MB. Copyright © 2021 Han and Lory. 2021 Han and Lory. https://creativecommons.org/licenses/by/4.0/ This content is distributed under the terms of the Creative Commons Attribution 4.0 International license.

Another *rpoS* ligation product was enriched at the adjacent region of the *ybiE* gene that has been recently shown to encode a small hydrophobic peptide (∼2 kDa) in an intergenic region between *ybiC* and *ybiJ* (35) (Fig. 4A). However, the analysis of the sequences in these *rpoS* chimeric transcripts showed that majority of these are antisense transcripts of YbiE, created by ligation of the 3′ OH of *rpoS* to 5′ of antisense transcripts of YbiE; we refer to this sRNA as asYbiE.

We monitored the expression of sAspA and asYbiE in LB (Luria-Bertani) rich medium by Northern blotting of total RNAs isolated from E. coli cultures at different growth phases. The levels of sAspA gradually increase at the stationary phase as ∼110 nt in size, with a concomitant increase of a smaller, ∼80-nt species. The asYbiE transcript is ∼350 nt in size in mid-log and stationary phase (Fig. 4B). The small size of asYbiE (∼100 nt) is detected only at the stationary phase with a weak signal, though overall it is hardly detectable under the LB medium condition without using a multicopy plasmid harboring the intergenic region between *ybiC* and *ybiJ* gene (Fig. 4B; see pIG_*ybiCJ*).

In order to examine the effect of the sRNAs on RpoS regulation in E. coli, we employed a plasmid which is able to express both an sRNA gene and the *rpoS^Ec^*::*lacZ* translational reporter gene fusion with different inducible promoters (P_BAD_ for sRNA and P_tac_ for *rpoS^Ec^*::*lacZ* gene, Fig. 4C). We first cloned the sequences encompassing the 5′ UTR and the codons for the first 80 amino acids of the E. coli *rpoS* gene into the plasmid pKH24::*lacZ* to create plasmid pKH24-*rpoS^Ec^*::*lacZ*. We then cloned each sRNA gene into this vector, to enable induction of its expression by the addition of arabinose, and introduced both of the constructs into E. coli Δ*lacIY*Δ*araCD*. We monitored the activity of the fusion when each sRNA (asYbiE or sAspA) was overexpressed. The previously identified sRNAs, DsrA and MgrR (24), were used as the positive and negative regulators, respectively, and as expected, they showed the predicted increase and decrease of β-galactosidase activity, respectively. The β-galactosidase activity was significantly reduced in the strain overexpressing asYbiE RNA, while in a strain over expressing the sAspA RNA, no effect was observed compared to the negative control (Fig. 4C). We also monitored the level of endogenous RpoS, using E. coli RpoS antibody, when asYbiE or sAspA was overexpressed. Consistent with the previous results obtained from the β-galactosidase activity assay, the Western blot analysis showed that overexpression of asYbiE led to a reduction of the endogenous RpoS protein levels (Fig. 4D). However, overexpression of sAspA had no effect on RpoS protein levels, though a possible RNA-RNA interaction between the 3′ UTR region of *rpoS* mRNA and sAspA can be identified (Fig. S2C). We predicted the likely base-pairing interactions between the asYbiE and the *rpoS* 5′ UTR sequence using IntaRNA or the RNA secondary structure of the chimeric RNA with the algorithm of the CLC Genomics Workbench package. The most likely interaction of asYbiE with its target is at the Shine-Dalgarno sequence of the *rpoS* mRNA (Fig. 4E), suggesting that the reduction of β-galactosidase activity of *rpoS*::*lacZ* and the RpoS protein is caused by interfering with initiation of translation by asYbiE, and therefore, it directly represses *rpoS* expression. In contrast, we cannot at this time assign a regulatory role for sAspA although potential base-pairing at the termination sequence of the coding region of *rpoS* could be computationally determined (Fig. S2C). Therefore, sAspA could be regulating other mRNAs, and the interaction detected by rGRIL-seq could represent a regulatory mechanism, where the *rpoS* mRNA is sequestering (“sponging”) the sAspA, reversing the effect of this sRNA on its other targets.

### sRNAs regulating P. aeruginosa *rpoS*.

**FIG 5:**
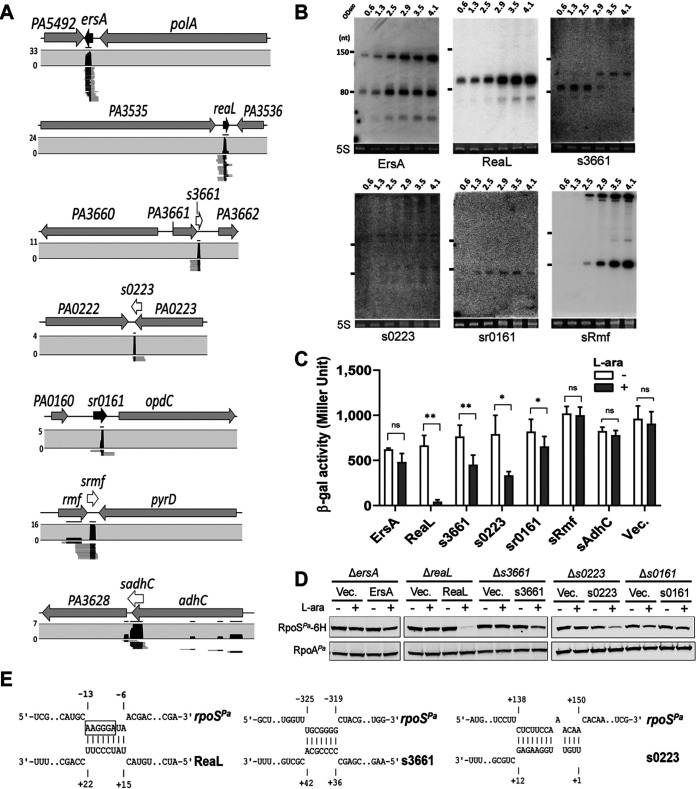
rGRIL-seq in P. aeruginosa identifies sRNAs interacting with *rpoS* mRNA. (A) The genomic locations of 7 different sRNAs found in *rpoS* chimeras by rGRIL-seq, ranked within the top 5 detected in exponential and stationary phase. The sRNAs annotated in previous studies are represented as black arrows while the sRNAs annotated in this study are represented as white arrows. (B) Northern blot analysis of six *rpoS*-interacting small transcripts (ErsA, ReaL, s3661, s0223, sr0161, and sRmf) in LB medium. 5S rRNAs stained with SYBR green were used as a loading control. (C) The effect of overexpression of the 7 sRNAs on the *rpoS^Pa^*::*lacZ* reporter. β-Galactosidase activity was measured in P. aeruginosa when each sRNA was overexpressed (gray) or not (white). The plasmid (Vec.) lacking an sRNA gene was used as a negative control. Data are shown as mean ± SD for three biological replicates. Statistical comparisons were performed using Student’s *t* test. *, *P* ≤ 0.05; **, *P* ≤ 0.01; ns, not significant. (D) Western blot for C-terminal 6×His-tagged RpoS levels in cells overexpressing sRNAs (ReaL, s3661, ErsA, s0223, and sr0161). (E) Base-pairing of ReaL (left), s3661 (middle), and s0223 (right) with *rpoS*. For the interaction between ReaL and the *rpoS* mRNA, the Shine-Dalgarno sequence of *rpoS* is boxed.

**FIG 6:**
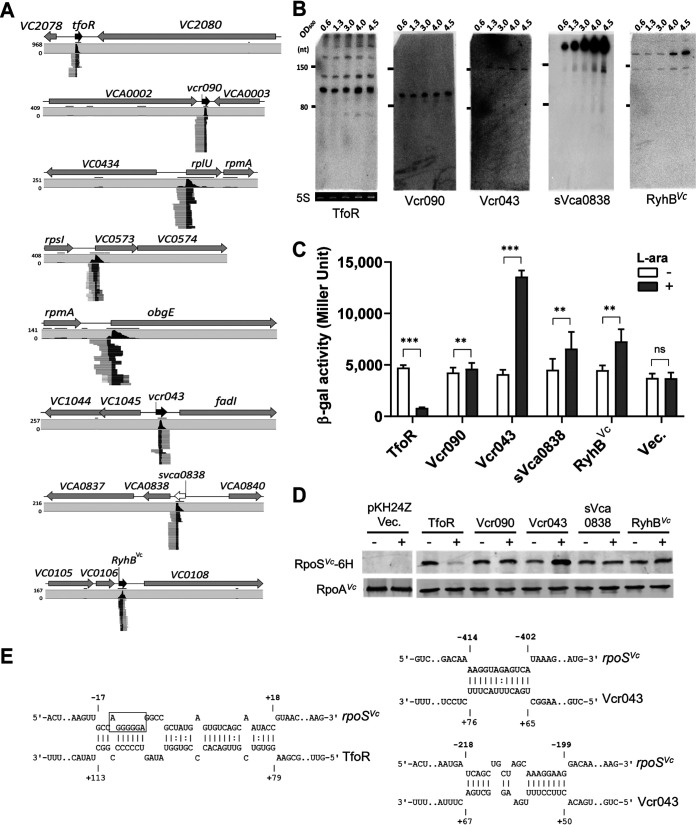
rGRIL-seq in V. cholerae identifies sRNAs interacting with the *rpoS* mRNA. (A) The genomic locations of a total of 8 different sRNAs found in chimeras with *rpoS*, ranked within the top 5 identified in exponential and stationary phase. (B) Northern blotting of 5 *rpoS*-interacting transcripts (TfoR, Vcr090, Vcr043, sVca0838, and RyhB*^Vc^*) in LB medium. 5S rRNAs stained with SYBR were used as a loading control, and the same membrane was used for deprobing and subsequent rehybridizing. (C) The effect of overexpression of the 5 sRNAs on the *rpoS^Vc^*::*lacZ* reporter. β-Galactosidase activity was measured in V. cholerae when each sRNA was overexpressed (gray) or not (white). The plasmid (Vec.) lacking the sRNA gene was used as a negative control. Data are shown as mean ± SD for four biological replicates. Statistical comparisons were performed using Student’s *t* test. **, *P* ≤ 0.01; ***, *P* ≤ 0.001; ns, not significant. (D) Western blotting for C-terminal 6×His-tagged RpoS levels in cells overexpressing sRNAs (TfoR, Vcr090, Vcr043, sVca0838, and RyhB*^Vc^*). The plasmid (pKH24Z/Vec.) expressing RpoS without the C-terminal 6×His and lacking the sRNA gene was used as a negative control. (E) Base-pairing between TfoR and *rpoS* (left) and the two sites of interaction between Vcr043 and *rpoS* (right).

When applying the rGRIL-seq method toward identifying sRNA regulators of P. aeruginosa *rpoS*, the number of chimeric reads from enriched samples was substantially lower than that with E. coli or V. cholerae. We recovered 296 sequencing reads in exponential phase and 3,467 reads in stationary phase, representing ∼31-fold less in exponential phase and ∼4-fold less in stationary phase compared to E. coli and ∼28-fold less in exponential phase and ∼3-fold less in stationary phase compared to V. cholerae (Table S1A). This poor recovery of *rpoS*-chimeric reads was accompanied by a substantial enrichment for chimeras containing rRNAs (Fig. 3B). They are likely created by a nonspecific ligation between random fragments of rRNAs and *rpoS* mRNA. The chimeras, generated by preferential base-pairing at regulatory sites, allow the sequencing reads to be visualized as peaks when mapped on the genome in the graphic display (Fig. 4A, Fig. 5A, and Fig. 6A and Fig. S3, S5, and S7). In contrast, nonspecific ligation products to rRNA are randomly distributed over the length of the gene rather than being enriched at a specific location (example shown in Fig. S4).

10.1128/mBio.03608-20.4 FIG S3 The enriched location of the 6 sRNA-*rpoS* chimeric reads on the MG1655 genome (left) and *rpoS* mRNA locus (red). Download FIG S3, PDF file, 0.5 MB. Copyright © 2021 Han and Lory. 2021 Han and Lory. https://creativecommons.org/licenses/by/4.0/ This content is distributed under the terms of the Creative Commons Attribution 4.0 International license.

10.1128/mBio.03608-20.5 FIG S4 Example of a false-positive rGRIL-seq result involving a highly expressed gene. Broad enrichment of rRNA-*rpoS* chimeras (yellow) seen over the entire length of the rRNA transcript formed by nonspecific ligation. Download FIG S4, PDF file, 0.03 MB. Copyright © 2021 Han and Lory. 2021 Han and Lory. https://creativecommons.org/licenses/by/4.0/ This content is distributed under the terms of the Creative Commons Attribution 4.0 International license.

10.1128/mBio.03608-20.6 FIG S5 The enriched location of the 7 sRNA-*rpoS* chimeric reads on the P. aeruginosa PAO1 genome (left) and *rpoS* mRNA locus (red). Download FIG S5, PDF file, 0.4 MB. Copyright © 2021 Han and Lory. 2021 Han and Lory. https://creativecommons.org/licenses/by/4.0/ This content is distributed under the terms of the Creative Commons Attribution 4.0 International license.

In our analysis of the P. aeruginosa chimeric reads, we identified a total of 7 different transcripts within the top 5 *rpoS*-chimeric RNAs (Table 1B and Fig. 5A). In spite of poor enrichment for the chimeras using the *rpoS*-complementary capture oligonucleotides, among the mapped sequences we found the ReaL sRNA (ranked at top no. 3 in Ex. and no. 2 in St., Table 1B), which has been previously reported as a negative regulator of *rpoS* expression in P. aeruginosa (36). This suggests that the our rGRIL-seq data may have other small RNA regulators for the *rpoS* mRNA but with lower numbers of chimeric reads. In our analysis of the 7 sRNA-*rpoS* chimeras, the most abundant *rpoS*-chimeric RNAs, in both exponential and stationary phase, contained ErsA, which has been previously shown to negatively regulate AlgC and OrpD and positively regulate AmrZ (37–39). Four sRNA-*rpoS* chimeric reads (s3661, s0223, sRmf, and sAdhC) were enriched near the stop codon or the 3′ UTR of mRNAs (*PA3661*, *PA0223*, *Rmf*, and *AdhC*, respectively; Fig. 5A), suggesting that these sRNAs represent the class of sRNAs derived by processing of mRNAs or internal starts within the coding sequences analogous to the sAspA RNA in E. coli. However, unlike the sAspA-*rpoS* sequences, none of these sRNA-*rpoS* chimeras showed enrichment of the reads at the 3′ region of the *rpoS* gene in P. aeruginosa (Fig. S5). In order to determine whether the candidate sRNAs can be identified in bacterial cells, we performed Northern blotting with total RNA isolated from P. aeruginosa PAO1 at different growth stages (Fig. 5B), and 6 of 7 total RNA transcripts were detected. With the exception of sAdhC, we were able to detect 3 transcripts (s3661, s0223, and sRmf) when they were probed with the radiolabeled oligonucleotides designed to bind to the 3′ region of the mRNAs (Fig. 5B and Fig. S6). The observation of the s3661, s0223, and sRmf small-size transcripts suggests that they are all derived from the 3′ UTR and all contain the rho-independent terminators.

10.1128/mBio.03608-20.7 FIG S6 The 3′-derived sRNA identified from rGRIL-seq in P. aeruginosa PAO1. The stop codon is represented as lowercase and underlined, and the rho-independent terminator is represented with filled arrows and “T.” The sRNA sequence is indicated with the empty arrow. The sequences in gray indicate the corresponding position of the oligonucleotide probe that was used in Northern blotting. Download FIG S6, PDF file, 0.01 MB. Copyright © 2021 Han and Lory. 2021 Han and Lory. https://creativecommons.org/licenses/by/4.0/ This content is distributed under the terms of the Creative Commons Attribution 4.0 International license.

10.1128/mBio.03608-20.8 FIG S7 The enriched location of the top 8 chimeric reads on the V. cholerae C6706 genome (left) and at the *rpoS* mRNA locus (red). Download FIG S7, PDF file, 0.5 MB. Copyright © 2021 Han and Lory. 2021 Han and Lory. https://creativecommons.org/licenses/by/4.0/ This content is distributed under the terms of the Creative Commons Attribution 4.0 International license.

We next constructed strains that allowed us to monitor the regulatory effect of overexpression of the putative sRNAs on *rpoS*. Based on the Northern blot analysis and the sequence of the rho-independent terminator, we predicted the 5′ and 3′ ends of each RNA transcript and cloned the putative sRNA genes into an arabinose-inducible expression plasmid (pKH6). We transformed each sRNA expression construct into P. aeruginosa carrying either the *rpoS^Pa^*::*lacZ* reporter or the gene for *rpoS^Pa^*::6XHis codons, inserting them into a chromosomal site (ϕCTX), and induced their expression with arabinose (Fig. 5C). In the β-galactosidase assay, we found that the ReaL sRNA shows a strong reduction in enzymatic activity of the reporter, in agreement with the previous report (36). Additionally, overexpression of four small RNAs (sr0161, s0223, s3661, and ErsA) also showed a moderate reduction of β-galactosidase activity compared with the control strain carrying the empty plasmid vector, suggesting that they might also function as negative regulators of RpoS. We further tested the effects of overexpression of these selected sRNAs on expression of the RpoS protein, using the C-terminal 6×His-tagged RpoS with a Western blot analysis probing with an antibody to the tag (Fig. 5D). In all cases, a similar reduction in RpoS*^Pa^*::6×His expression was detected as seen in the β-galactosidase assay, further indicating that the sRNAs identified by rGRIL-seq are negative regulators of *rpoS* mRNA, leading to a reduction in the levels of the RpoS protein. Focusing on the three sRNAs (ReaL, s3661, and s0223) showing the most pronounced reduction of RpoS following their overexpression, we predicted their base-pairing with *rpoS* mRNA using the IntaRNA algorithm (34) and analysis of the secondary RNA structure of the sRNA-*rpoS* chimeric RNA (Fig. 5E). The predictions show that ReaL base pairs with the Shine-Dalgarno sequence of *rpoS* mRNA as shown in the previous report (36). However, s3661 and s0223 were predicted to base-pair mRNA at regions −325 to −319 for s3661 and +138 to +150 for s0223 from the translational start codon of *rpoS*, suggesting the possibility of the presence of other regulatory sites and different mechanisms to repress the *rpoS* translation by these sRNAs.

### sRNAs regulating V. cholerae *rpoS*.

To identify sRNA regulators of *rpoS* in V. cholerae C6706 by rGRIL-seq, we mapped all *rpoS* chimeric reads on the two chromosomes (referred to as Ch1 and Ch2) of V. cholerae N16961, since the genes have been well annotated and are closely related to the C6706 strain. Applying the rGRIL-seq procedure to V. cholerae, we recovered a similar number of chimeric reads as in E. coli (Table S1A): on average, 8,331 reads in late exponential phase and 10,132 reads in early stationary phase. Mapping all of the *rpoS* chimeric reads onto the genome, within the top 5, we identified a total of 8 different transcripts under our two experimental growth conditions (Fig. 3C and Table 1C). Among them, 5 *rpoS* chimeric transcripts were created in the intergenic regions (TfoR, Vcr090, Vcr043, sVca0838, and RyhB*^Vc^*), while three were created with the transcripts from start codon regions of mRNAs (*rplU*, *Vc0573*, and *obgE*; Fig. 6A). The inspection of sequences at the ligation junctions revealed that 5 (TfoR, Vcr090, rplU, Vcr043, and RyhB*^Vc^*) of the 8 transcripts formed chimeric products with fragments near the 5′ UTR of *rpoS* (Fig. S7), suggesting that they are directly regulating either the expression of *rpoS* mRNA stability or its translation. The other three chimeras (sVc0573, sVca0838, and *obgE*) were created by ligation to *rpoS* sequences near the *rpoS* stop codon and the 3′ UTR of the mRNA.

We assessed the expression of the 5 sRNAs (TfoR, Vcr090, Vcr043, sVca0838, and RyhB*^Vc^*), transcribed from intergenic regions by Northern blot analysis in rich medium (LB) over several growth phases (Fig. 6B). All sRNAs were detected; they varied in size from ∼90 nt (Vcr090) to ∼350 nt (sVca0838) during the growth in LB; some showed accumulation in the stationary phase (sVca0838 and RyhB*^Vc^*) while others (TfoR, Vcr090, and Vcr043) were found at similar levels through all growth phases. TfoR is an ∼100-nt sRNA which has been shown previously to target and positively regulate *tfoX* mRNA, encoding the key regulator of natural competence and type VI secretion in V. cholerae (40–42). We observed a major ∼100-nt TfoR RNA in the Northern blot experiment, while two more minor TfoR transcripts (∼130 nt and ∼250 nt) were also detected. Vcr090 and Vcr043 were previously reported as ∼90- and ∼160-nt sRNAs, respectively, in wild-type V. cholerae C6706 (43), and they were detected as well at similar sizes in the Northern blot assay. Based on the size of sVca0838 in the Northern blot and the presence of the rho-independent terminator sequence, this new sVca0838 sRNA is transcribed as a 346-nt RNA. We also detected RyhB*^Vc^* at the corresponding size of 214 nt; its function ranges from control of iron homeostasis to regulation of biofilm formation (44).

In order to assess the effect of each sRNA on regulation of RpoS expression in V. cholerae, we employed the same plasmid vector (pKH24::*lacZ*) previously used in E. coli for measuring β-galactosidase activity. We amplified the sequences encompassing the 5′ UTR and codons for the first 80 amino acids of the V. cholerae *rpoS* gene to construct the plasmid pKH24-*rpoS^Vc^*::*lacZ*. After inserting the 5 sRNA genes individually into the vector, we introduced each plasmid into the V. cholerae C6706 *recA*-lacZ* mutant cell. In the analysis of β-galactosidase activity in strains with the *lacZ* reporter, the most pronounced effect was observed following overexpression of TfoR, which significantly reduced the expression of the *rpoS-lacZ* fusion protein (∼6-fold). In contrast, the overexpression of three of the sRNAs (Vcr043, sVca0838, and RyhB*^Vc^*) resulted in a modest induction of the *rpoS^Vc^*::*lacZ* translation (∼3-fold for Vcr043, ∼1.4-fold for sVca0838, and ∼1.6-fold for RyhB*^Vc^*). In order to monitor the level of the RpoS protein in V. cholerae, we constructed the plasmid pKH24-*rpoS^Vc^*::*6H* expressing RpoS*^Vc^* fused to the His tag at its C terminus and then inserted each of the 5 sRNA genes into this vector. Following induction with arabinose, TfoR overexpression resulted in a significant reduction of RpoS::6×His as measured by Western immunoblotting. In contrast, Vcr043 overexpression led to an increase in RpoS::6×His levels (Fig. 6D). Based on the mapping of the chimeric RNAs with the TfoR and Vcr043 on the *rpoS* transcript (Fig. S7), combined with predictions of likely interactions using IntaRNA, base-pairing models were generated and are shown in Fig. 6E. The most likely base-pairing site of TfoR on the *rpoS* transcript is near the initiation codon (−17 to +18). This predicted interaction suggests that TfoR sRNA negatively regulates RpoS expression by base-pairing with the Shine-Dalgarno sequences and/or the start codon. The predicted base paring model between TfoR and *rpoS^Vc^* shows that the region on the sRNA involved in base-pairing is located between nucleotides +79 and +113. This region differs from that predicted for the interaction of TfoR with *tfoX* where the base-pairing region is at the 5′ end of TfoR, located between nucleotides +1 and +61 (45). In the predicted base-pairing model for Vcr043 and the *rpoS* mRNA, two possible base-pairing regions can be identified (Fig. 6E) and both are found at two different sites at the 5′ UTR (−414 to −402 and −218 to −199) on the *rpoS* mRNA. In summary, our rGRIL-seq data along with these results show that in V. cholerae, TfoR functions as a negative regulator of *rpoS* in addition to a positive regulator of *tfoX*, while Vcr043 functions as a positive regulator of *rpoS* expression.

## DISCUSSION

In the work presented here, we describe a modification of the previously developed proximity ligation method (GRIL-seq) for studying sRNA interactions with their targets. The main difference in the current rGRIL-seq approach and previous GRIL-seq is in the enrichment step, where in this case chimeras are captured using oligonucleotides complementary to the mRNA target sequences, thus allowing the identification of sRNA regulators of specific mRNAs. Combined with GRIL-seq, rGRIL-seq allows for a more comprehensive probing of sRNA regulons, potentially leading to the identification of new sRNAs that could not be predicted using computational tools or through the analysis of the transcriptomes due to their low abundance. Currently, a list of sRNAs transcribed in intergenic regions can be readily derived by combining transcriptome sequencing (RNA-seq) with computational analyses. However, recent findings that sRNA regulators can be also generated by fragmentation of mRNAs or transcription initiation within coding sequences (3, 33) make it difficult to analyze their roles in posttranscriptional regulatory networks. Therefore, rGRIL-seq represents another method where the identity of an sRNA is revealed by becoming a substrate for *in vivo* ligation to an mRNA target and does not depend on any prior knowledge of its origin. Additionally, rGRIL-seq represents a useful approach for studying the regulatory activities of multiple sRNAs in controlling the expression from a single mRNA target in response to different environmental conditions. Finally, rGRIL-seq has an added advantage over other experimental methods developed for detecting sRNA-mRNA interactions such as RIL-seq and Hi-GRIL-seq (9, 38) because the complementary oligonucleotide enrichment step allows the detection of interactions between sRNA and their target mRNAs in low abundance.

**FIG 7:**
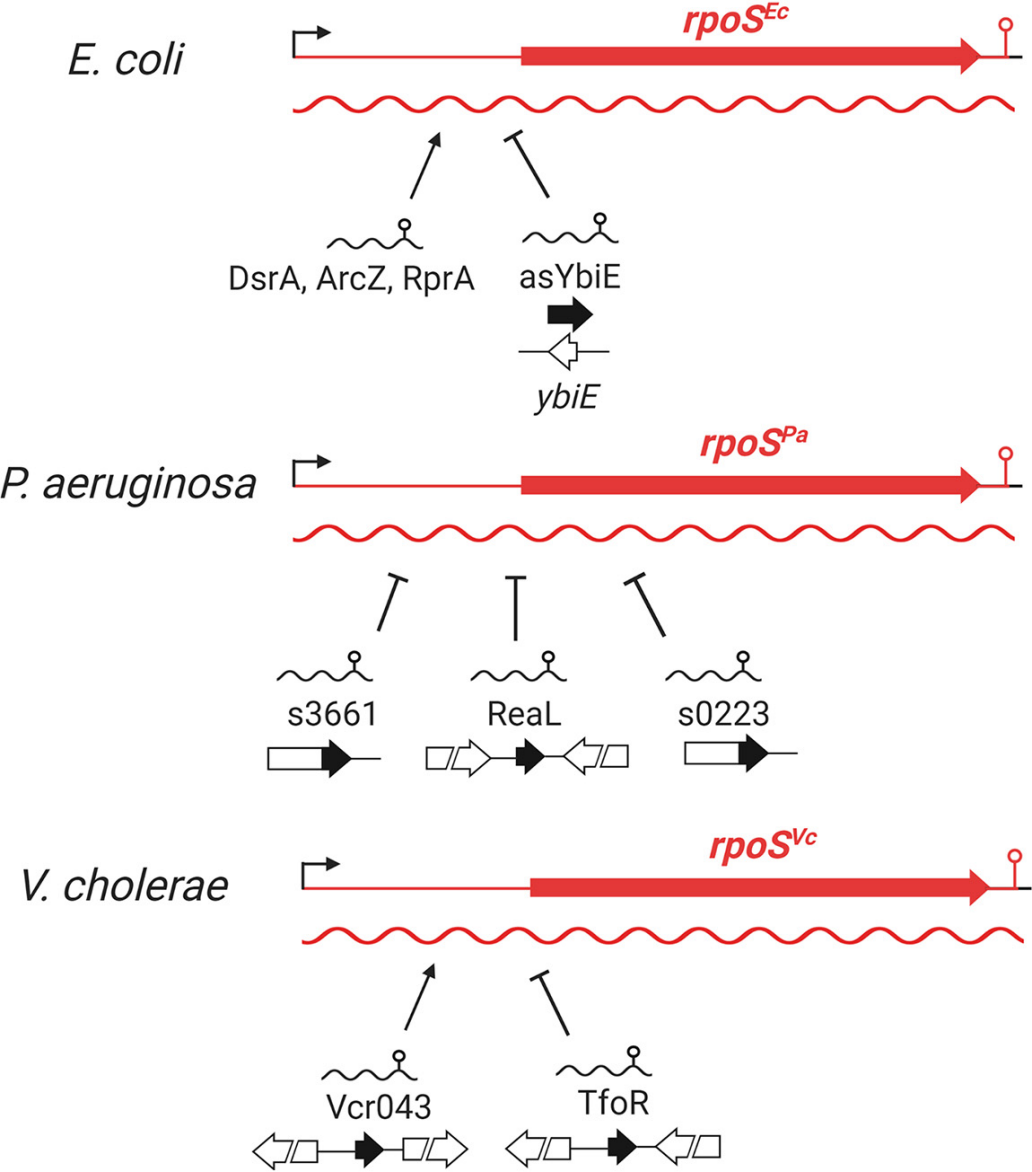
Summary of the posttranscriptional regulatory inputs by various sRNAs on the expression of *rpoS* of E. coli, P. aeruginosa, and V. cholerae identified by rGRIL-seq. In E. coli, asYbiE functions as a negative RNA regulator of *rpoS*, while three known sRNAs (DsrA, ArcZ, and RprA) are positive regulators. In P. aeruginosa, ReaL is a negative regulator of *rpoS* targeting the region containing the Shine-Dalgarno sequence. In addition, sRNAs s3661 and s0223 generated from 3′ regions of the *PA3661* and *PA0223* mRNA, respectively, act as negative regulators of *rpoS*. In V. cholerae, the expression of *rpoS* is regulated positively by Vcr043 and negatively by TfoR.

Here, we applied rGRIL-seq to identify sRNA regulators of expression of the stationary-phase sigma factor RpoS in E. coli, P. aeruginosa, and V. cholerae. This approach allowed us to validate rGRIL-seq in an organism (E. coli) where posttranscriptional regulation of *rpoS* expression has been extensively studied. Additionally, we probed sRNA regulation of *rpoS* in two organisms (P. aeruginosa and V. cholerae) that, because of their distinct natural environments, have likely evolved unique stress survival strategies. The summary of the distinct regulatory mechanisms for the control of *rpoS* expression in the three organisms studied is shown schematically in Fig. 7.

### Positive regulators of RpoS expression.

Using rGRIL-seq in E. coli, we confirmed that the three previously characterized sRNA activators (ArcZ, DsrA, and RprA) interact with *rpoS* mRNA and create RpoS chimeric RNAs in a reaction catalyzed by RNA ligase. We applied this method to P. aeruginosa and V. cholerae and identified Vcr043 sRNA as an sRNA activator of V. cholerae *rpoS*, while finding no activators of P. aeruginosa *rpoS* under our conditions.

We noted that the formation of RNA chimeras seems to require the cleavage of the 5′ UTR of *rpoS* mRNA or the sRNA, or both. When the activator sRNAs interact with the internal region of the long 5′ UTR, the cleavage of the 5′ UTR appears to be a prerequisite to the formation of the chimeric RNAs by the coexpressed T4 RNA ligase. For example, in our analysis of the DsrA-*rpoS* chimeric transcripts, the major ligation reaction in the DsrA-*rpoS* chimeric RNAs occurred in the internal region (−107 nt relative to translation initiation site) of the previously characterized DsrA base paring site (−120 to −90 nt) (19), suggesting that base-pairing, concomitant cleavage, and ligation are important factors to create chimeric RNAs under *in vivo* proximity ligation conditions. Indeed, it has been reported that base-pairing of DsrA with the 5′ UTR of *rpoS* leads to a concomitant cleavage of both the 5′ UTR of *rpoS* and DsrA by RNase III (46, 47). We have previously also observed this form of chimeric RNAs in RyhB*^Ec^*-*shiA* chimeric RNAs in E. coli (13). In the rGRIL-seq analysis in P. aeruginosa, we were unable to find activators of *rpoS* under the experimental condition used to grow the bacteria. It is not clear at present whether these sRNA activators are not found in this organism or we are unable to detect them due to the absence of the RNase-mediated cleavage event in the 5′ UTR as shown in E. coli DsrA, which is necessary to create the chimeric RNAs between the activator sRNA and *rpoS* in P. aeruginosa. Additionally, further studies are needed to determine whether rGRIL-seq is capable of identifying the activators such as RydC that base-pair in the middle of the 5′ UTR of *cfa* transcript, stabilizing it by inhibiting the cleavage from RNase E (48).

In the rGRIL-seq analysis of sRNA-*rpoS* interactions in V. cholerae, we identified Vcr043 as a strong activating sRNA of *rpoS* expression (Fig. 6C and D). Our prediction of a Vcr043-*rpoS* duplex based on the two forms of chimeric RNAs suggested two base-pairing sites for Vcr043 on the 5′ UTR of *rpoS*. The Vcr043-*rpoS* chimeric RNAs identified in rGRIL-seq also appear to be created following the cleavage of the 5′ UTR of *rpoS* and ligation to Vcr043 as previously shown in E. coli DsrA RNA. However, it remains to be determined whether base-pairings of the sRNA to both sites on the *rpoS* transcript are necessary for the activation of expression and what RNase is involved in the cleavage of the 5′ UTR. The Vcr043 was previously identified in a transcriptomic analysis in V. cholerae (43). Interestingly, its gene is located upstream of the VC1045 gene encoding RNA polymerase sigma factor 70 and oppositely transcribed to the VC1045 gene. In our analysis of their transcription start sites (TSSs) based on transcriptomic data, the distance between their TSSs is 25 bp and their predicted promoters are partially overlapped. However, further studies are needed to establish whether the housekeeping sigma factor 70 (σ^70^) is also regulated with the sRNA activator to control sigma factor S (σ^S^) in V. cholerae.

### Negative regulators of RpoS expression.

Using rGRIL-seq in E. coli, we identified the sRNA asYbiE as a repressor of *rpoS* translation. Resembling the mode of action of many negative sRNA regulators studied previously, asYbiE appears to base-pair with the translation initiation site of *rpoS* and represses its translation. Though the rGRIL-seq shows a relatively high number of asYbiE-*rpoS* chimeric reads (ranked no. 5 in total), it seems to be expressed at a low level in rich media (Fig. 4B). The asYbiE is an antisense RNA of *ybiE* which encodes a small peptide (2 kDa), YbiE, composed of 19 hydrophobic amino acids (35). Since YbiE is known to be expressed in both exponential and stationary growth phases in rich media (35), asYbiE seems to be negatively regulated by the expression of YbiE. In P. aeruginosa, we confirmed the previously characterized ReaL sRNA as a repressor of *rpoS*. In addition, we also identified two 3′-derived sRNAs (s3661 and s0223) as repressors of *rpoS* expression. As seen previously (36), overexpression of ReaL shows a significant repression of *rpoS*, while two 3′-derived sRNAs demonstrated only moderate repression. The predicted location of the base-pairing regions of the 3′-derived repressor sRNAs, at the upstream region of the 5′ UTR (for s3661) and in the coding region of *rpoS* for s0223, is uncommon for sRNAs interfering with the initiation of translation. This suggests that s3661 and s0223 may negatively regulate *rpoS* expression by mechanisms differing from the canonical action of sRNAs targeting the Shine-Dalgarno sequence and the start of translation.

Using rGRIL-seq in V. cholerae, we identified the new regulatory function of the TfoR sRNA, as a repressor of *rpoS*. TfoR sRNA has been known as a positive regulator of *tfoX* by base-pairing with the 5′ UTR of *tfoX* mRNA and activating the translation (45). RpoS and TfoX are known to be required for natural transformation of V. cholerae (49). In the light of our finding that TfoR regulates the two mRNAs in an opposite manner, many questions remain about the physiological relevance of reciprocal expression of natural competence and stress response by V. cholerae.

In addition to finding positive and negative regulators of *rpoS*, we also observed a few featured interactions at the 3′ region of the *rpoS* transcript whose regulatory consequence were not addressed in this study. In E. coli, sAspA RNA seems to interact with the 3′ UTR of *rpoS*, while sVca0838 seems to interact with the 3′ coding region of *rpoS* in V. cholerae (Fig. S3 and S7). However, when examining the effect of these sRNAs when overexpressed on RpoS protein levels, they appear to have no effect on *rpoS* translation. It is still an open question whether the transcripts originating from the 3′ region of *rpoS* might function as new 3′-derived sRNAs sequestering (sponging) sAspA and sVca0838.

Our work presented here also highlights two general concepts applicable to sRNA regulation. First, as illustrated with *rpoS*, sRNAs and their targets can follow an evolutionary pathway unique to each organism reflecting the adaption to their specific environmental niches. Second, the expression of sRNAs is likely regulated and under the control of various transcription factors, highlighting the need to consider the interplay of transcriptional and posttranscriptional inputs when elucidating the workings of regulatory networks. The analysis of sRNA-mRNA interactions should therefore take full advantage of complementary tools such as GRIL-seq and rGRIL-seq to provide a comprehensive picture of the consequences of regulatory events leading to effective response to changing environmental conditions.

## MATERIALS AND METHODS

### Oligonucleotides and plasmids.

The oligonucleotides and plasmids used in this study are listed in Table S2A and B in the supplemental material, respectively. The construction of recombinant plasmids was carried out using an In-Fusion cloning kit (Clontech, catalog no. 639648), and T4 DNA ligase when required. To construct the dual expression plasmids (pKH24::Z and pKH24::6H), the linear vector pKH24XS (Amp^r^) was amplified from pBAD24 by PCR with R_pKH24Xblvec and F_KH24SphIvec, adding new restriction enzyme sites, XbaI and SphI, at the site following the P_BAD_ promoter site. The amplified linear vector (pKH24XS) was circularized by T4 DNA ligase and transformed into E. coli competent cells (Clontech, catalog no. 636763). The plasmid pKH24XS was purified and linearized by the restriction enzyme NsiI. The *lacZ* gene and 6×His tag sequence with the *lacI*^q^ gene were amplified by PCR from plasmids pmini-Ptac::*lacZ* and pmini-Ptac::6His (13), respectively, with a primer pair (F_DelNsil_lacI/R_Nsil_*lacZ*_6H). These PCR products were ligated with the previous pKH24XS (NsiI digested) using the In-Fusion cloning kit to construct pKH24::Z and pKH24::6H. The ligated plasmids were transformed into competent E. coli, and the plasmids were purified to construct E. coli or V. cholerae *rpoS* translational fusions. To construct the *rpoS*::*lacZ* translational fusion vectors (pKH24-rpoS*^Ec^*^/^*^Vc^*::*lacZ*), the sequences from the transcription start site (TSS) to the 80th amino acid codon of the E. coli and V. cholerae *rpoS* gene were amplified from E. coli MG1655 and V. cholerae C6706 genomes with primer pairs F_ERI_ecrpoS+80aa/R_Hd3_ecrpoS+80aa and F_ERI_vcrpoS+80aa/R_Hd3_vcrpoS+80aa, respectively. The plasmid pKH24::*lacZ* was linearized with restriction enzymes (EcoRI and HindIII), and the *rpoS* genes from each of the three bacterial species, previously obtained by PCR, were inserted by ligation. To construct the His-tagged fusion vectors (pKH24-rpoS*^Ec^*^/^*^Vc^*::6H), the sequence from the TSS to the sequence corresponding to *rpoS^Ec/Vc^* up to the stop codon was amplified from E. coli and V. cholerae chromosomal DNA. The PCR products were cloned into the linearized pKH24::6H (EcoRI/HindIII). The ligated vectors were introduced into competent E. coli by electroporation, and the plasmids were purified and used to clone each sRNA gene into these vectors (pKH24-rpoS*^Ec/Vc^*::*lacZ* and rpoS*^Vc^*::6H). Each E. coli or V. cholerae sRNA gene was amplified with their corresponding primer pairs (Table S2A), and the vectors were digested with XbaI and SphI. The PCR products were ligated with the vectors and transformed into competent E. coli. All sRNA sequences used for expression are listed in Table S2C. All plasmids carrying sRNA and *rpoS^Ec/Vc^*::*lacZ* (or *rpoS^Vc^*::*6H*) genes were purified and electroporated into E. coli MG1655 Δ*lacIY* Δ*araCD* and the V. cholerae C6706 *recA*-lacZ* mutant for measurements of β-galactosidase assays or Western blotting. To construct the vector for expressing P. aeruginosa sRNAs, their corresponding sRNA genes were amplified from the P. aeruginosa PAO1 genome and cloned into pKH6 (Gm^r^) as described previously (13). For chromosomal expression of *rpoS^Pa^*::*lacZ* fusion or the 6×His-tagged proteins, the two different lengths of *rpoS^Pa^* genes were amplified as carried out in E. coli and cloned into the digested plasmids (EcoRI/HindIII; pPtac-miniCTX::*lacZ* or pPtac-miniCTX::*6H*) as performed previously (13).

10.1128/mBio.03608-20.10 TABLE S2 Oligonucleotides (A) and strains or plasmids (B) used in this study and sRNA sequence (C) cloned into the sRNA expression vector. Download Table S2, PDF file, 0.2 MB. Copyright © 2021 Han and Lory. 2021 Han and Lory. https://creativecommons.org/licenses/by/4.0/ This content is distributed under the terms of the Creative Commons Attribution 4.0 International license.

### Bacterial strains.

The list of bacterial strains created for this study is provided in Table S2B. E. coli MG1655, P. aeruginosa PAO1, and V. cholerae C6706 are referred to as the wild-type strains. When required, they were used for a mutant construction. In order to prevent catabolism of arabinose and X-Gal (5-bromo-4-chloro-3-indolyl-β-d-galactopyranoside) in β-galactosidase assays, E. coli MG1655 Δ*araDC* Δ*lacAI* was engineered from the MG1655 Δ*lacAI* strain lacking the genes *lacAYZI* using the lambda Red recombineering system as described previously (50). Briefly, the genes *araDABC* were disrupted by homologous recombination with a PCR product consisting of a kanamycin resistance gene (Kan^r^) flanked by 40-bp regions homologous to *araD* and *araC* genes on the E. coli chromosome. For engineering a chromosomal deletion of a gene in P. aeruginosa, the plasmid pEXG2 was used as described previously (51). For the β-galactosidase assays and plasmid stability in V. cholerae, the spontaneous mutant of *lacZ* (referred to as lacZ*) in the C6706 *recA*^−^ strain was used to prevent catabolism of X-Gal. For rGRIL-seq, E. coli MG1655, P. aeruginosa PAO1, and V. cholerae C6706 *recA*-lacZ* strains were used.

### β-Galactosidase assays.

Assays of β-galactosidase activity in E. coli and V. cholerae were carried out in the same manner. Each overnight culture of E. coli or V. cholerae containing the plasmid pKH24-rpoS*^Ec^*^/^*^Vc^*::*lacZ*/sRNA was grown in LB with 50 μg/ml carbenicillin. The overnight culture was diluted to an optical density at 600 nm (OD_600_) of ∼0.02 in 1 ml LB containing the same concentration of antibiotics and then grown to an OD_600_ of ∼1.0. To express the sRNA and *rpoS* mRNA, l-arabinose and IPTG (isopropyl-β-d-thiogalactopyranoside) were added to the final concentrations of 0.02% and 50 μM, respectively. β-Galactosidase activity was measured using 50 μl of cells after 3.3 h of induction with l-arabinose and IPTG. Miller units were calculated as described previously (52). For β-galactosidase assays in P. aeruginosa, an overnight culture of P. aeruginosa (pPtac-miniCTX-rpoS::*lacZ*) carrying either the empty vector (pKH6) or pKH6-sRNA was grown in LB with 37.5 μg/ml tetracycline and 75 μg/ml gentamicin. The overnight culture was diluted as done for E. coli, and l-arabinose and IPTG were added to the final concentrations of 0.2% and 100 μM, respectively. β-Galactosidase activity was measured using 50 μl of cells after 3.5 h of induction with l-arabinose and IPTG.

### Western blot analysis.

To prepare the protein samples for Western blot analysis, E. coli strains containing the plasmids with the *rpoS* gene and expressing different sRNAs (pKH24-rpoS*^Ec^*::*lacZ*/sRNA) were grown as described for the β-galactosidase assay. After 2.3 h of induction, cells from a 300-μl culture were collected by centrifugation (15,000 × *g*; for 1 min), and the total protein samples were prepared as described previously (13). To monitor the levels of endogenous RpoS*^Ec^*, monoclonal antibody against E. coli RpoS (BioLegend, catalog no. 663104) was used according to the manufacturer’s instructions. For the protein analysis in V. cholerae, the strains carrying pKH24-rpoS*^Vc^*::6H were grown as described for the β-galactosidase assay. The preparation of protein samples from P. aeruginosa and Western blotting was described previously (13). To monitor the C-terminal His-tagged RpoS protein levels in V. cholerae and P. aeruginosa, the His tag antibody (GenScript, catalog no. A00186) was used according to the manufacturer’s instruction.

### rGRIL-seq.

The rGRIL-seq was carried out as described for GRIL-seq (13) with the following modifications. Cultures of E. coli MG1655, P. aeruginosa PAO1, or V. cholerae C6706 strains carrying the plasmid pKH13-*t4rnl1* were grown overnight aerobically with shaking at 37°C, in LB (Luria-Bertani) broth with carbenicillin (for E. coli and V. cholerae, 50 μg/ml; for P. aeruginosa, 150 μg/ml). The overnight cultures were diluted into the same medium to an OD_600_ of 0.01. For induction of the T4 RNA ligase, IPTG was added to 1 mM when the culture reached a low (OD_600_ of ∼0.4) or high (OD_600_ of ∼3.5) cell density. After induction for E. coli *and* V. cholerae (40 min) and for P. aeruginosa (60 min), cells from 0.6 ml of each culture were used for total RNA isolation by the hot-phenol-chloroform extraction method. The cultures were directly treated with a phenol mixture at 65°C, which contained 0.1 volume of RNA extraction buffer (20 mM sodium acetate, 0.5% SDS, 1 mM EDTA, pH 5.2) and the same volume (0.6 ml) of phenol saturated with the RNA extraction buffer. The phenol-cell mixture was vigorously mixed and incubated for 10 min with an intermittent vigorous mix. After centrifugation (21,000 × *g*) for 10 min at 4°C, the aqueous phase was isolated and mixed with 0.55 ml of phenol-chloroform. After a vigorous mix, the two phases were separated by centrifugation for 5 min. The aqueous phase was mixed with 0.45 ml of chloroform to remove the residual phenol in the aqueous phase. After a vigorous mix, the two phases were separated by centrifugation for 5 min, and the aqueous phase containing purified total RNAs (final volume of 0.4 ml) was mixed with 2.5 volumes of ethanol (1 ml) and stored at −80°C. The total RNA was collected by centrifugation (10 min, 21,000 × *g*, 4°C) and washed with 70% ethanol (0.7 ml). The dried RNA pellets were dissolved with nuclease-free water (40 μl), and 12 μg of total RNA was used for the enrichment of chimeric RNAs. For the enrichment of *rpoS* chimeric RNAs, 4 multiple DNA oligonucleotides were designed and 20 pmol (5 pmol for each) of oligonucleotides was mixed and used in the enrichment step of rGRIL-seq. All steps for the enrichment and the following precipitation of the *rpoS* chimeric RNAs were carried out as described for GRIL-seq (13). The enriched chimeric RNAs were precipitated at −80°C, collected by centrifugation (10 min, 21,000 × *g*, 4°C), and washed with 70% ethanol (0.5 ml). The enriched RNAs were dissolved in 13 μl of nuclease-free water, and 100 ng of enriched RNAs was used for RNA library preparation using the NEBNext Ultra Directional RNA library prep kit (NEB, catalog no. E7420s). A total of 11 PCR cycles was performed for amplification of the library, and then the library was purified twice with AMPure XP (Beckman Coulter, catalog no. A63880) according to the NEBNext Ultra Directional RNA library prep kit. The library derived from this RNA was subjected to sequencing on a NextSeq500 instrument (Illumina) with 150-bp paired-end reading.

### Data analysis.

Data analysis for rGRIL-seq was performed using CLC Genomics Workbench 7.0 as used for GRIL-seq (13); the detailed protocol is described in Text S1 in the supplemental material.

10.1128/mBio.03608-20.1 TEXT S1 Data analysis of rGRIL-seq using CLC Genomics Workbench 7.0. Download Text S1, PDF file, 0.05 MB. Copyright © 2021 Han and Lory. 2021 Han and Lory. https://creativecommons.org/licenses/by/4.0/ This content is distributed under the terms of the Creative Commons Attribution 4.0 International license.

### RNA isolation and Northern blot analysis.

Overnight cultures of the bacteria grown at 37°C were diluted in LB to an initial OD_600_ of ∼0.02. Cell growth was monitored at different time points, and total RNA was isolated by the hot-phenol method as described above for rGRIL-seq. For Northern blotting, total RNA (10 μg) was mixed with RNA loading buffer II (Ambion) and denatured at 95°C for 3 min. The RNA was fractionated on a 5% polyacrylamide-7 M urea gel in 1× Tris-borate-EDTA (TBE). The fractionated RNAs were electrotransferred to a Hybond N+ membrane (GE Healthcare) and cross-linked on the membrane using the Stratalinker UV cross-linker on the autocrosslink setting (2 times, 120,000 μJ/cm^2^). After prehybridization with Rapid-hyb buffer (GE Healthcare), a ^32^P-labeled oligonucleotide probe was added, and after overnight hybridization at 43°C, the membrane was washed with 2× SSC-0.1% SDS and 0.5× SSC-0.1% SDS buffer (1× SSC is 0.15 M NaCl plus 0.015 M sodium citrate). If required for deprobing, the blots were incubated in hot 0.1% (wt/vol) SDS solution twice for 15 min with gentle agitation and then the residual SDS was removed with distilled water. The signal was visualized on Typhoon FLA 7000 (GE Healthcare). Each oligonucleotide used to prepare a radioactive probe is listed in Table S2A.

### *In silico* prediction of base-pairing between sRNA and target RNAs.

IntaRNA software 2.0 (34) was used to predict base-pairing interactions between sRNA and target RNAs. In the seed parameters, the default for the minimum number of base pairs in the seed region was set as 7. When required, it was set as 6. For the prediction of secondary RNA structure of the chimeric RNA, generally a single chimeric RNA sequence was extracted for the chimeric DNA reads, and the secondary RNA structure algorithm of CLC Genomics Workbench 7.0 was used according to the manufacturer’s instructions.
