# Supplementary material for: Toward a Comprehensive Analysis of Posttranscriptional Regulatory Networks: a New Tool for the Identification of Small RNA Regulators of Specific mRNAs
Source: mBio. 2021 Feb 23;12(1):e03608-20. doi: 10.1128/mBio.03608-20 (PMC8545128; doi:10.1128/mBio.03608-20)
Supplement: FIG S1 [file mbio.03608-20-sf001.pdf]

Fig. S1

A

```
rpoS_Pa_PAO1        -------MALKKEGPEFDHDDEVL-LLE--PGIMLDESSADEQPSPRATPKATTSFSSKQ   50
rpoS_Ec_MG1655      --MSQNTLKVHDLNEDAEFDENGVEVFDEKA-------LVEQEPSDN------DLAEEEL   45
rpoS_Vc_C6706       MSVSNTVTKV----EEFDFEDEALEVLETDAELTSDEELVAVEGASE------DV----R   46
                              :     : :.::: : :::           .  : : .

rpoS_Pa_PAO1        HKHIDYTRALDATQLYLNEIGFSPLLTPEEEVHFARLAQKGDPAGRKRMIESNLRLVVKI  110
rpoS_Ec_MG1655      LSQGATQRVLDATQLYLGEIGYSPLLTAEEEVYFARRALRGDVASRRRMIESNLRLVVKI  105
rpoS_Vc_C6706       EEFDASAKSLDATQMYLSEIGFSPLLTAEEEVLYARRALRGDEAARKRMIESNLRLVVKI  106
                     .     : *****:**.***:***** **** :** * :** *.*:**************

rpoS_Pa_PAO1        ARRYVNRGLSLLDLIEEGNLGLIRAVEKFDPERGFRFSTYATWWIRQTIERAIMNQTRTI  170
rpoS_Ec_MG1655      ARRYGNRGLALLDLIEEGNLGLIRAVEKFDPERGFRFSTYATWWIRQTIERAIMNQTRTI  165
rpoS_Vc_C6706       SRRYSNRGLALLDLIEEGNLGLIRAVEKFDPERGFRFSTYATWWIRQTIERALMNQTRTI  166
                    :*** ****:****************************************:*******

rpoS_Pa_PAO1        RLPIHVVKELNVYLRAARELTHKLDHEPSPEEIANLLEKPVAEVKRMLGLNERVTSVDVS  230
rpoS_Ec_MG1655      RLPIHIVKELNVYLRTARELSHKLDHEPSAEEIAEQLDKPVDDVSRMLRLNERITSVDTP  225
rpoS_Vc_C6706       RLPIHVVKELNIYLRTARELSQRLDHEPTPEEIALELDRPVDDVTKMLRLNERISSVDTP  226
                    *****:*****:***:****:::*****: ****  *::** :*.:** ****::***.

rpoS_Pa_PAO1        LGPDSDKTLLDTLTDDRPTDPCELLQDDDLSESIDQWLTELTDKQREVVIRRFGLRGHES  290
rpoS_Ec_MG1655      LGGDSEKALLDILADEKENGPEDTTQDDDMKQSIVKWLFELNAKQREVLARRFGLLGYEA  285
rpoS_Vc_C6706       IGGDGDKALLDILPDSHNADPEFSTQDDDIRESLLNWLDELNPKQKEVLARRFGLLGYEP  286
                    :* *.:*:*** * *.:  .*    ****: :*: :** **. **:**: ***** *:*

rpoS_Pa_PAO1        STLEEVGQEIGLTRERVRQIQVEALKRLREILEKNGLSSDALFQ-----   334
rpoS_Ec_MG1655      ATLEDVGREIGLTRERVRQIQVEGLRRLREILQTQGLNIEALFRE----   330
rpoS_Vc_C6706       STLEEVGREINLTRERVRQIQVEGLRRLREILVKQGLNMEALFNVEYDN   335
                    :***:**:**.***********.*:****** .:**. :***.
```

B

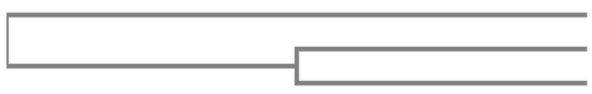

**rpoS_Pa**	**100**	**67**	**67**
**rpoS_Ec**	**67**	**100**	**74**
**rpoS_Vc**	**67**	**74**	**100**

C

```
rpoS_Ec_MG1655      UUCGGGUGAACAGAGUGCUAACAAAAUGUUGCCGAACAACAAGCCAACUGCGACCACGGU  -508
rpoS_Pa_PAO1        ------------------------------------------------------------  -365
rpoS_Vc_C6706       ------------GUCGGUAAACAAAAUGUUA----------------------------  -478

rpoS_Ec_MG1655      CACAGCGCCUGUAACGGUACCAACAGCAAGCACAACCGAGCCGACUGUCAGCAGUACAUC  -448
rpoS_Pa_PAO1        ------------------------------------------------------------  -365
rpoS_Vc_C6706       --------AUGUUAAUGUAGCAAAAGCUAAACCUUCG----------------------  -449

rpoS_Ec_MG1655      AACCAGUACGCCUAUCUCCACCUGGCGCUGGCCGACUGAGGGCAAAGUGAUCGAAACCUU  -388
rpoS_Pa_PAO1        ------------------------------------------------------------  -365
rpoS_Vc_C6706       ----GAUGAGAAGAUAGCGAAGUGGCUAUGGCCGACAAAAGGUAGAGUCAUAAAGAACUU  -393

rpoS_Ec_MG1655      UGGCGCUUCUGAGGGGGGCAACAAGGGGAUUGAUAUCGCAGGCAGCAAAGGACAGGCAAU  -328
rpoS_Pa_PAO1        ------------------------------------------------------------  -365
rpoS_Vc_C6706       UUCUGCGGGCGAUCAAGGCAACAAAGGGAUAGACAUUGCUGGCCAACGUGGUCAGGCUGU  -333

rpoS_Ec_MG1655      UAUCGCGACCGCAGAUGGCCGCGUUGUUUAUGCUGGUAACGCGCUGCGCGGCUACGGUAA  -268
rpoS_Pa_PAO1        ----GCUGCGUCUGGUGGGACCGUUGUAUACGCCGGUAGUGGUUUGCGGGGCUACGGCGA  -310
rpoS_Vc_C6706       CGUUGCUACUGCAGAUGGAACCGUAGUGUAUUCGGGCAACGCAUUACGUGGUUAUGGCAA  -273
                        **  *  * * ***   *** ** **  * ** *  *   * ** ** ** **  *

rpoS_Ec_MG1655      UCUGAUUAUCAUCAAACAUAAUGAUGAUUACCUGAGUGCCUACGCCCAUAACGACACAAU  -208
rpoS_Pa_PAO1        GUUGGUCAUCAUCAAACACAACGAGACCUACGUGAGUGCCUACGGUCACAACCGCAGGCU  -250
rpoS_Vc_C6706       CCUAAUCAUCAUAAAACAUAAUGAGCACUAUUUAAGUGCCUAUGCCCACAAUGAUCAGCU  -213
                      *  * ***** ***** ** **    **  * ******** *  ** **       *

rpoS_Ec_MG1655      GCUGGUCCGGGAACAACAAGAAGUUAAGGCGGGGCAAAAAAUAGCGACCAUGGGUAGCAC  -150
rpoS_Pa_PAO1        GCUGGUGCGGGAAGGGCAACAGGUCAAGGUAGGGCAAUCGAUUGCCGAGAUGGGCUCCAC  -190
rpoS_Vc_C6706       GCUAGCAAAGGAAGGACAAACUGUGCAAGCAGGCCAGAAGAUCGCCACCAUGGGAAGUUC  -153
                    *** *    ****   ***   **  * *  ** **    ** **    *****     *

rpoS_Ec_MG1655      CGGAACCAGUUCAACACGCUUGCAUUUUGAAAUUCGUUACAAGGGGAAAUCCGUAAACCC   -88
rpoS_Pa_PAO1        AGGAACCGAUCGGGUGAAGCUGCACUUCGAGAUUCGCCGCCAGGGUAAGCCUGUCGAUCC  -130
rpoS_Vc_C6706       UGGUACCAACAGCGUACGCUUGCACUUUGAGAUCCGUUAUCAAGGGAAGUCAGUGAAUCC   -93
                     ** ***              **** ** ** ** **     * ** **  * **  * **

rpoS_Ec_MG1655      GCUGCGUUAUUUGCCGCAGCGAUAAAU-CGGCGG------AAC-CAGGCUUUU-GCU-UG  -38
rpoS_Pa_PAO1        ACUGCAAUAUUUGCCACGUCGCUGACCG-GGAGUUCGCCCGCCCACAUCAUGUAGGUGAG  -71
rpoS_Vc_C6706       UAAACGCUACUUACCUUAACUCAUAUUGCUAAGGUCAUACUGC-AAAGGUUGUAAAUCGC  -34
                        *  ** ** **    *    *       *         *       * *   *

rpoS_Ec_MG1655      AAUGUUCCGUCAAGGGAUCACGGGU--AGGAGCC-----ACCUU----------------  -1
rpoS_Pa_PAO1        CGGGUCCGG--GCGUGUCCAGCGGGAAAGGAAUCGCCCGGGCUUGAGUCGAACUCAUGCA  -13
rpoS_Vc_C6706       AAUGACUCGCAAAGUUGCCAGGGGG--AGG---C-----CGCU----------------  -1
                       *    *    *    **  **   ***   *       **

rpoS_Ec_MG1655      ------------         -1
rpoS_Pa_PAO1        AGGGAUAACGAC         -1
rpoS_Vc_C6706       ------------         -1
```

D

```
rpoS_Ec_MG1655      AUGAGUC--AG-AAUACGCU---GAAAGUUCAUGAUUUAAAUGAAGAUGCGGAAUUUGAU   54
rpoS_Pa_PAO1        --------------AUGGCACUCAA-----------AAAAGAAGGGCCGGAGUUUGAC   33
rpoS_Vc_C6706       AUGAGUGUCAGCAAUACCGUAACCAAAGUAG-----------AAGAGUUCGAUUUUGAA   48
                                  *       **                ***     ** *****

rpoS_Ec_MG1655      GAGAACGGAGUUGAGGUUUUUGACGAAAAGGCCUUAGUAGAACAGGAACCCAGUGAUAAC  114
rpoS_Pa_PAO1        CACGAUG---AUGAAGUGCUCCUCCUGGAGCCC-----------GGCAUCAUGCUGGAC   78
rpoS_Vc_C6706       GAUGAAGCACUGGAAGUGCUAGAAACUGAUGCC---------GAGCUCACCAGUGAUGAA   99
                     *  * *     ** **  *       *  **                **     *

rpoS_Ec_MG1655      GAUUUGGCCGAAGAGGA-----------A-------------C-------------UGU  136
rpoS_Pa_PAO1        GAGUCGUCUGCCGACGAGCAGCCUUCUCCCCGGGCAACUCCAAAAGCCACCACUUCCUUC  138
rpoS_Vc_C6706       GAAUUAGUUGCUGUUGA-----------AGGGGCAAGUGAAGA-------------CGU  134
                    ** *     *  *  **

rpoS_Ec_MG1655      UAUCGCAGGGA-GCCA-----------CACAGCGUGUGUUGGACGCGACUCAGCUUUAC  183
rpoS_Pa_PAO1        UCUUCCAAACAACACAAGCACAUCGACUACACGCGCGCGUUGGACGCAACGCAGCUGUAU  198
rpoS_Vc_C6706       UCGUGAAGAGU-UUGAU-------GCUUCUGCGAAAAGUCUUGAUGCGACCCAGAUGUAU  186
                    *     *        *                 *       * ** ** ** *** * **

rpoS_Ec_MG1655      CUUGGUGAGAUUGGUUAUUCACCACUGUUAACGGCCGAAGAAGAAGUUUAUUUUGCGCGU  243
rpoS_Pa_PAO1        CUCAACGAAAUCGGUUUCUCGCCCCUGUUGACGCCCGAAGAGGAAGUCCACUUCGCUCGU  258
rpoS_Vc_C6706       CUCAGCGAAAUUGGUUUUUCACCGCUCCUUACUGCCGAAGAAGAAGUGCUUUAUGCUCGU  246
                    **    ** ** ****  ** ** **  * **  ******* *****    *  ** ***

rpoS_Ec_MG1655      CGCGCACUGCGUGGAGAUGUCGCCUCUCGCCGCCGGAUGAUCGAGAGUAACUUGCGUCUG  303
rpoS_Pa_PAO1        CUGGCGCAGAAGGGCGAUCCCGCUGGUCGGAAGCGGAUGAUCGAGAGCAACCUGCGGUUG  318
rpoS_Vc_C6706       CGUGCCUUACGUGGUGAUGAAGCCGCACGUAAACGCAUGAUUGAAAGUAACUUGCGUCUG  306
                    *  **       ** ***   **    **    ** ***** ** ** *** ****  **

rpoS_Ec_MG1655      GUGGUAAAAAUUGCCCGCCGUUAUGGCAAUCGUGGUCUGGCGUUGCUGGACCUUAUCGAA  363
rpoS_Pa_PAO1        GUGGUGAAGAUCGCCCGGCGCUAUGUCAAUCGCGGACUGUCCCUGCUCGACCUGAUCGAG  378
rpoS_Vc_C6706       GUGGUAAAAAUUUCACGCCGUUACAGCAACCGAGGAUUAGCACUGCUCGAUCUGAUUGAA  366
                    ***** ** **  * ** ** **   *** ** **  *  *  **** ** ** ** **

rpoS_Ec_MG1655      GAGGGCAACCUGGGGCUGAUCCGCGCGGUAGAGAAGUUUGACCCGGAACGUGGUUUCCGC  423
rpoS_Pa_PAO1        GAAGGCAACCUAGGCCUGAUCCGCGCCGUGGAGAAGUUCGAUCCGGAGCGCGGAUUCCGG  438
rpoS_Vc_C6706       GAAGGUAAUCUUGGUCUGAUCCGUGCGGUUGAGAAAUUCGAUCCAGAACGCGGUUUCCGC  426
                    ** ** ** ** ** ******** ** ** ***** ** ** ** ** ** ** *****

rpoS_Ec_MG1655      CUGGAUAAGCCAGUUGAUGACGUCAGCCGUAUGCUUCGUCUUAACGAGCGCAUUACCUCG  663
rpoS_Pa_PAO1        CUGGAGAAGCCGGUCGCCGAGGUCAAGCGCAUGCUCGGCCUGAACGAACGGGUGACUUCG  678
rpoS_Vc_C6706       UUAGACCGACCUGUCGAUGAUGUCACUAAGAUGCUGCGUCUUAACGAACGGAUCAGCUCA  666
                     * **    ** ** *  ** ****     *****  * ** ***** **  * *  **

rpoS_Ec_MG1655      GUAGACACCCCGCUGGGUGGUGAUUCCGAAAAAGCGUUGCUGGACAUCCUGGCCGAUGAA  723
rpoS_Pa_PAO1        GUAGACGUCUCUCUUGGUCCGGACUCGGACAAGACCCUGCUGGAUACGCUCACCGACGAU  738
rpoS_Vc_C6706       GUGGAUACGCCAAUUGGUGGGGAUGGAGAUAAGGCACUGCUGGAUAUUUGCCAGACUCU  726
                    ** **     *  * ***   **    ** **  *  ******* *   *  * **

rpoS_Ec_MG1655      AAAGAGAACGGUCCGGAAGAUACCACGCAAGAUGACGAUAUGAAGCAGAGCAUCGUCAAA  783
rpoS_Pa_PAO1        CGCCCCACCGAUCCGUGCGAGCUGCUGCAGGAUGACGAUCUCAGCGAAAGCAUCGACCAG  798
rpoS_Vc_C6706       CACAAUGCCGAUCCUGAGUUUUCAACUCAAGAUGAUGACAUUCGUGAAUCGCUGCUCAAC  786
                           ** ***            ** ***** **  *    *    *  * *

rpoS_Ec_MG1655      UGGCUGUUCGAGCUGAACGCCAAACAGCGUGAAGUGCUGGCACGUCGAUUCGGUUUGCUG  843
rpoS_Pa_PAO1        UGGCUGACGGAACUCACCGACAAGCAGCGUGAGGUGGUGAUUCGCCGCUUCGGCUUGCGC  858
rpoS_Vc_C6706       UGGUUGGAUGAACUUAAUCCAAAGCAAAAAGAAGUGCUUGCGCGUCGCUUUGGGCUUCUU  846
                    *** **   ** ** *    ** **    ** *** *    ** ** ** **  * *

rpoS_Ec_MG1655      GGGUACGAAGCGGCAACACUGGAAGAUGUAGGUCGUGAAAUUGGCCUCACCCGUGAACGU  903
rpoS_Pa_PAO1        GGUCACGAAAGCAGCACGCUGGAAGAGGUCGGCCAGGAAAUCGGCCUGACCCGCGAGCGG  918
rpoS_Vc_C6706       GGCUAUGAACCAUCGACCUUGGAAGAAGUGGGUCGUGAGAUCAAUCUCACUCGUGAGCGU  906
                    **  * ***      **  ******* ** ** *  ** **    ** ** ** ** **

rpoS_Ec_MG1655      GUUCGCCAGAUUCAGGUUGAAGGCCUGCGCCGUUUGCGCGAAAUCCUGCAAACGCAGGGG  963
rpoS_Pa_PAO1        GUUCGUCAGAUCCAGGUCGAGGCGCUGAAGCGCCUGCGGGAGAUUCUGGAGAAGAAUGGC  978
rpoS_Vc_C6706       GUUCGCCAAAUCCAAGUGGAAGGUCUACGUCGUCUGCGUGAGAUUUUGGUGAAACAAGGU  966
                    ***** ** ** ** ** ** *  **    **  **** ** **  **   *  * **

rpoS_Ec_MG1655      CUGAAUAUCGAAGCGCUGUUCCGCGAGUAA------------  993
rpoS_Pa_PAO1        CUGUCGAGUGACGCGCUGUUCCAGUGA--------------  1005
rpoS_Vc_C6706       UUGAAUAUGGAAGCGCUGUUUAACGUCGAAUACGACAACUAA  1008
                     **   *  ** ********
```

E

```
rpoS_Ec_MG1655      GUAAGCAUCUGUCAGA--------AAGGCCAGUCUC----AAGCGAGGCUGGCCUUUUCU  1041
rpoS_Pa_PAO1        --CGGAAAACCUUAGACCCACUGAAG--------ACCCGGCG-UUCGCCGGGUUUUUU--  1052
rpoS_Vc_C6706       ------UUUUUCCAGACUCAUCCAAAACUAAGGCACCCGGUGGGGUGCCUUGUUGUUUCU  1062
                           ***       *         *         * *  *   ***

rpoS_Ec_MG1655      GUG 1044
rpoS_Pa_PAO1        --- 1052
rpoS_Vc_C6706       --- 1062
```

F

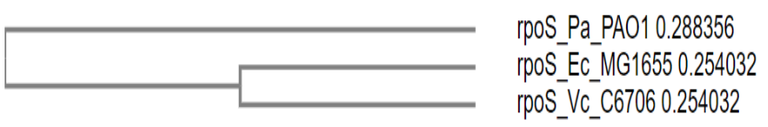

**rpoS_Pa**	**100**	**62**	**55**
**rpoS_Ec**	**62**	**100**	**61**
**rpoS_Vc**	**55**	**61**	**100**

G

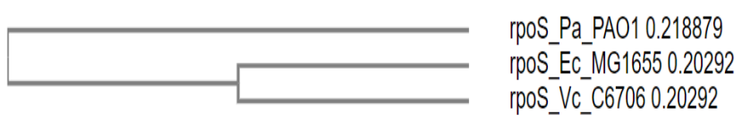

**rpoS_Pa**	**100**	**68**	**62**
**rpoS_Ec**	**68**	**100**	**68**
**rpoS_Vc**	**62**	**68**	**100**

H

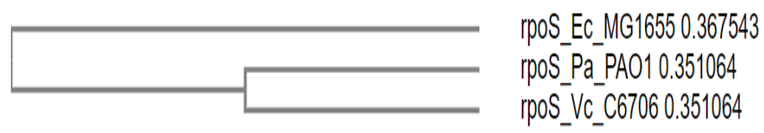

**rpoS_Pa**	**100**	**61**	**63**
**rpoS_Ec**	**61**	**100**	**60**
**rpoS_Vc**	**63**	**60**	**100**
