# Supplementary material for: Toward a Comprehensive Analysis of Posttranscriptional Regulatory Networks: a New Tool for the Identification of Small RNA Regulators of Specific mRNAs
Source: mBio. 2021 Feb 23;12(1):e03608-20. doi: 10.1128/mBio.03608-20 (PMC8545128; doi:10.1128/mBio.03608-20)
Supplement: TABLE S1 [file mbio.03608-20-st001.pdf]

Table S1.

A. Chimeric *rpoS* mRNAs obtained from cells expressing endogenous *rpoS*.

	Ex. (~0.4 OD_{600})			St. (~3.5 OD_{600})		
**rpoS_*Ec***	**ex1**	**ex2**	**ex3**	**st1**	**st2**	**st3**
**total reads**	77,434,318	46,043,954	75,601,580	72,057,280	54,786,860	54,736,782
**rpoS mapped reads**	7,618,741	4,466,841	7,988,455	13,987,794	9,842,498	8,811,043
**rpoS chimeric reads**	10,711	7,296	9,784	17,402	13,984	10,770
**enrichment efficiency**	9.8	9.7	10.6	19.4	18	16.1
**chimeric RNA/rpoS and rpoS chimeric (%)**	0.14	0.16	0.12	0.12	0.14	0.12
**rpoS_*Pa***	**ex1**	**ex2**	**ex3**	**st1**	**st2**	**st3**
**total reads**	44,197,308	65,387,832	53,303,996	75,128,940	64,072,978	70,051,682
**rpoS mapped reads**	586,737	1,178,578	430,460	6,150,198	5,119,909	5,180,730
**rpoS chimeric reads**	232	471	186	3,980	3,327	3,095
**enrichment efficiency (%)**	1.3	1.8	0.8	8.2	8	7.4
**chimeric RNA/rpoS and rpoS chimeric (%)**	0.04	0.04	0.04	0.06	0.06	0.06
**rpoS_*Vc***	**ex1**	**ex2**	**ex3**	**st1**	**st2**	**st3**
**total reads**	40,829,958	54,739,274	47,446,860	43,434,928	41,414,266	49,910,962
**rpoS mapped reads**	2,131,465	3,137,459	2,621,012	8,588,928	10,110,842	9,390,392
**rpoS chimeric reads**	7,864	9,782	7,347	13,750	7,645	9,002
**enrichment efficiency**	5.2	5.7	5.5	19.8	24.4	18.8
**chimeric RNA/rpoS and rpoS chimeric (%)**	0.37	0.31	0.28	0.16	0.08	0.1

B. RNAs interacting with *rpoS* mRNA in *E. coli*, *P. aeruginosa*, and *V. cholerae* identified by rGRIL-seq.

rpoS_Ec

Ex.

Rank	Gene Name	Average maximal coverage (>35)	Mapping location of chimeras	flanking genes	
				5'	3'
1	arcZ	998	sRNA	elbB	arcB
2	dsrA	780	sRNA	yodD	yedP
3	mgrR	606	sRNA	mgtS	dgcZ
4	saspA	126	mRNA3'	dcuA	fxsA
5	asybiE	111	antisense RNA	ybiC	ybiJ
6	gcvB	95	sRNA	gcvA	ygdI

7	cyaR	82	sRNA	yegQ	ogrK
8	fimI	44	mRNA5'	fimA	fimC
9	gadY	41	sRNA	gadW	gadX
10	spot42	35	sRNA	polA	yihA

St.

Rank	Gene Name	Average maximal coverage (>35)	Mapping location of chimeras	flanking genes	
				5'	3'
1	arcZ	1601	sRNA	elbB	arcB
2	mgrR	1115	sRNA	mgtS	dgcZ
3	dsrA	916	sRNA	yodD	yedP
4	gadY	369	sRNA	gadW	gadX
5	asybiE	315	antisense RNA	ybiC	ybiJ
6	ryeB	280	sRNA	pphA	yebY
7	saspA	245	mRNA3'	dcuA	fxsA
8	gadF	229	sRNA	yhiE	yhiU
9	yjbj	173	mRNA3'	dinF	zur
10	cpxQ	146	sRNA	cpxP	yiiP
11	aceK	116	mRNA5'	aceA	arpA
12	rprA	109	sRNA	ydiK	ydiL
13	tnaL	108	mRNA3'	trmE	tnaA
14	cyaR	75	sRNA	yegQ	ogrK
15	fimI	74	mRNA5'	fimA	fimC
16	malG	59	mRNA3'	xylE	malF
17	yfiA(3)	43	mRNA3'	b2596	pheL
18	sdsN	41	sRNA	sdiA	yecC
20	osmB	35	mRNA5'	yciH	yciT

rpoS_Pa

Ex.

Rank	Gene Name	Average maximal coverage (>35)	Mapping location of chimeras	flanking genes	
				5'	3'
1	esrA	12	sRNA	PA5492	PA5493
2	s3661	3	mRNA3'	PA3661	PA3665
3	reaL	2	sRNA	PA3535	PA3536
4	sr0161	2	sRNA	PA1060	opdP
5	s0223	2	mRNA3'	PA0222	PA0224

St.

Rank	Gene Name	Average maximal coverage (>35)	Mapping location of chimeras	flanking genes	
				5'	3'
1	esrA	24	sRNA	PA5492	PA5493
2	reaL	15	sRNA	PA3535	PA3536

3	srmF	9	mRNA3'	PA3049	PA3050
4	sadhC	7	mRNA3'	PA3628	PA3630
5	s3661	6	mRNA3'	PA3661	PA3665
6	ssrA	4	tmRNA	PA0826	PA0827
7	crcZ	4	sRNA	PA4726.1	PA4727
8	rnpB	3	housekeeping RNA	PA4421	PA4422
9	s0223	3	mRNA3'	PA4385	PA4387
10	groES	2	mRNA5'	PA4385	PA4387
11	cysN	2	mRNA5'	PA4441	PA4443
12	ssrS	2	6S RNA	PA5227	PA5228

rpoS_Vc

Ex.

Rank	Gene Name	Average maximal coverage (>35)	Mapping location of chimeras	flanking genes	
				5'	3'
1	tfoR	564	sRNA	vc2078	vc2080
2	vcr090	200	sRNA	vca0002	vca0003
3	rplU	177	mRNA5'	vc0434	vc0436
4	vc0573	134	mRNA5'	vc0572	vc0574
5	obgE	100	mRNA5'	vc0436	vc0438
6	rpsU	99	mRNA5'	vc0519	vc0521
7	vc0532	91	mRNA	vc0531	vc0533
8	vc2044-5	66	IGR	vc2044	vc2045
9	vca0832	61	mRNA5'	vca0831	vca0833
10	vc0894-6	61	IGR	vc0894	vc0896
11	vca0661	57	mRNA5'	vca0659	vca0662
12	vc0530	56	mRNA5'	vc0529	vc0531
13	vc0218	52	mRNA5'	vc0217	vc0217
14	vca1077-8	49	IGR	vca1077	vca1078
15	vc1996	47	mRNA5'	vc1995	vc1997
16	vc1821	46	mRNA3'	vc1820	vc1822
17	vc1262	35	mRNA5'	vc1261	vc1263
18	vc0518	35	mRNA5'	vc0517	vc0519

St.

Rank	Gene Name	Average maximal coverage (>35)	Mapping location of chimeras	flanking genes	
				5'	3'
1	vcr090	276	sRNA	vc2078	vc2079
2	vc0573	222	mRNA5'	vc0572	vc0574
3	vcr043	170	sRNA	vc1045	vc1046
4	svca0838	123	IGR	vca0838	vca0840
5	RyhBvc	98	sRNA	vc0106	vc0108

6	vc1996	88	mRNA5'	vc1995	vc1997
7	vc0489-90	82	sRNA	vc0489	vc0490
8	vc1502-3	63	IGR	vc1502	vc1503
9	obgE	61	mRNA3'	vc0436	vc0438
10	vc1060	60	mRNA5'	vc1059	vc1061
11	vc2221	57	mRNA5'	vct082	vc2222
12	vc2036	56	mRNA5'	vc2035	vc2037
13	vc0218	54	mRNA	vc0217	vc0217
14	vc0142a	49	mRNA5'	vc0142	vc0143
15	vca1077-8	45	IGR	vca1077	vca1078
16	vc1333-4	44	IGR	vc1333	vc1334
17	vca0661	41	mRNA5'	vca0660	vca0662
18	vc0150	40	mRNA5'	vc0149	vc0151
19	vc2260	37	mRNA	vc2259	vc2261

Table S3. Oligonucleotides used in this study.

Name	Sequence (5' to 3')	Used for
R_pKH24Xblvec	tctagaaacagtagagagttgc	cloning for pKH24XS
F_KH24SphIvec	aagcttggctgttttggc	cloning for pKH24XS
F_DelNsil lacI	cagcttatcatcgatgcaCTCTTCTGCTCCCGAAC	PCR for IacIq and lacZ (or 6His)
R_NsiI_lacZ_6H	caggcacattatgcataattaaccctcactaaagggaac	PCR for IacIq and lacZ (or 6His)
F_ERI_ecrpoS+80aa	CAATATAATGTGTG GAATTC TTCGGGTGAACAGAGTGC	cloning for pKH24-rpoS^{Ec}::lacZ
R_Hd3_ecrpoS+80aa	tgtaaaacgacggcAAGCTT CGCAAAATAAACTTCTTCTTC	cloning for pKH24-rpoS^{Ec}::lacZ
F_ERI_vcrpoS+80aa	CAATATAATGTGTG GAATTC GTCGGTAAACAAAATGTTAATG	cloning for pKH24-rpoS^{Vc}::lacZ (and -::6H)
R_Hd3_vcrpoS+80aa	tgtaaaacgacggcAAGCTT ATAAAGCACTTCTTCTTCGGC	cloning for pKH24-rpoS^{Vc}::lacZ
R_Hnd3_6H_vcrpoS	GTGGTGgccAAGCTT GTTGTCGTATTCGACGTTAAAC	cloning for pKH24-rpoS^{Vc}::6H
F_XbaI_asYbiE+1	CAACTCTCTACTGTT TCTAGA T ATTTCCCTGTCTGTTTGCCG	cloning for pKH24-rpoS^{Ec}::lacZ/asYbiE
R_sphI_pkh24_asYbiE	catccgccaaaacagcc gcatgc GCTTTCTGTCAGCAGTTAGCC	cloning for pKH24-rpoS^{Ec}::lacZ/asYbiE
F_XbaI_sAspA+1	CAACTCTCTACTGTT TCTAGA T AATCTGATGCACCCGGCTTA	cloning for pKH24-rpoS^{Ec}::lacZ/sAspA
R_sphI_pkh24_sAspA	catccgccaaaacagcc gcatgc GTTATTTTTAAGTTACTGCTC	cloning for pKH24-rpoS^{Ec}::lacZ/sAspA
F_XbaI_DsrA+1	CAACTCTCTACTGTT TCTAGA T AACACATCAGATTTCCTG	cloning for pKH24-rpoS^{Ec}::lacZ/DsrA
R_sphI_pkh24_DsrA	catccgccaaaacagcc gcatgc GGATATTCATGACTTCAG	cloning for pKH24-rpoS^{Ec}::lacZ/DsrA
F_XbaI_MgrR+1	CAACTCTCTACTGTT TCTAGA T aGATTCGTTATCAGTGC	cloning for pKH24-rpoS^{Ec}::lacZ/MgrR
R_sphI_pkh24_MgrR	catccgccaaaacagcc gcatgc GGGCCTGATTTTTATGAC	cloning for pKH24-rpoS^{Ec}::lacZ/MgrR
F_ERI_pa_rpoS+80aa	CAATATAATGTGTG GAATTC GCTGCGTCTGGTGGGAC	cloning for pPtac-miniCTX-rpoSPa::lacZ
R_Hd3_pa_rpoS+80aa	tgtaaaacgacggcAAGCTT CTCTTCGGGCGTCAACAGG	cloning for pPtac-miniCTX-rpoSPa::lacZ
R_Hd3_rpoS_ctxH6	GTGGTGgccAAGCTT CTGGAACAGCGCGTCACTCG	cloning for pPtac-miniCTX-rpoSPa::6H
F_XbI_pKH6ErsA+1	CAACTCTCTACTGTT TCTAGA T ACGAATGGCTTCTTGAGCC	cloning for pKH6-ErsA
R_Hnd3_pKH6ErsA+163	CCGCCAAAACAGCC AAGCTT CAAGAAGCGTTCGAGGAAG	cloning for pKH6-ErsA
F_xbaI_pkh6ReaL+1	CAACTCTCTACTGTT TCTAGA T ATCCAGCGCTGTACTATCC	cloning for pKH6-ReaL
R_Hnd3_pkh6ReaL	CCGCCAAAACAGCC AAGCTT CATTCGTCGCGGATTTGCAG	cloning for pKH6-ReaL
F_XbI_pKH6s3661+1	CAACTCTCTACTGTT TCTAGA T AAGCGCACCACGACGATTC	cloning for pKH6-s3661

R_Hnd3_pKH6 s3661+104	CCGCCAAAACAGCC AAGCTT GGCTGTTTGGCGCAGGAC	cloning for pKH6-s3661
F_XbaI_pKH6s 0223+1	CAACTCTCTACTGTT TCTAGA T ACTTCATCCTCCGCCG	cloning for pKH6-s0223
R_Hnd3_pKH6 s0223+229	CCGCCAAAACAGCC AAGCTT CTGAGCGCGCTGCGCGTC	cloning for pKH6-s0223
F_XbI_pKH6-sRmf+1	CAACTCTCTACTGTT TCTAGA T AACACGGATAGCACCGATTTC	cloning for pKH6-sRmf
R_Hnd3_pKH6-sRmf+115	CCGCCAAAACAGCC AAGCTT GACGCCATCGCCGCATTG	cloning for pKH6-sRmf
F_XbaI_pKH6s adhc+1	CAACTCTCTACTGTT TCTAGA T AAGGCGCAGAAGGGCGAG	cloning for pKH6-sadhC
R_Hnd3_pKH6 sadhc+140	CCGCCAAAACAGCC AAGCTT GGAATCCTTCACGTGAC	cloning for pKH6-sadhC
F_pKH6_XbaIs r0161	CAACTCTCTACTGTT TCTAGA T ACTTCCCGACGCCGAACTTTG	cloning for pKH6-s0161
R_pKH6_Hnd3 sr0161	CCGCCAAAACAGCC AAGCTT GGCGATCGCTCCCATGG	cloning for pKH6-s0161
F_BHI_Del_Ers A	CAGGTCGACTCTAGA GGATCC GGAGGCCCAGGCCTACATC	cloninig for pEXG2-ΔesA
R_Del_ErsA_fu s	GTTCGGGGTTCAAGTGAAAAACTTTTCCACGGCACC CTC	cloninig for pEXG2-ΔesA
F_Del_ErsA_fu s	GTGCCGTGGAAAAGTTTTTCACTTGAACCCCGAACTT CCC	cloninig for pEXG2-ΔesA
R_ERI_Del_Ers A	GGAAATTAATTAAGGTACCGAATTC AGGGCCTGGAAGTCGC	cloninig for pEXG2-ΔesA
F_BHI_Del_Re aL	CAGGTCGACTCTAGA GGATCC AGCTCGACGCACAGAGCAG	cloninig for pEXG2-ΔreaL
R_Del_ReaL-fus	CTGCAAATATGAGAAAAAGACCTAACTGCTCCTCTC GCACCCTCC	cloninig for pEXG2-ΔreaL
F_Del_ReaL-fus	GGAGGGTGCGAGAGGAGCAGTTAGGTCTTTTTCTCA TATTTGCAG	cloninig for pEXG2-ΔreaL
R_ERI_Del_Re aL	GAAATTAATTAAGGTACC GAATTC GGTTTCGGACCAGGGAGGTC	cloninig for pEXG2-ΔreaL
F_BHI_Del_s36 61	CAGGTCGACTCTAGA GGATCC CATTCGCACAATCCGTACG	cloninig for pEXG2-Δs3661
R_Del_s3661-fus	CAGGGGGAGAACAGCGTGTTAGCCGCAGGGCTGCTG C	cloninig for pEXG2-Δs3661
F_Del_s3661-fus	CCGCAGCAGCCCTGCGGCTAACACGCTGTTCTCCCC CTG	cloninig for pEXG2-Δs3661
R_ERI_Del_s36 61	GAAATTAATTAAGGTACC GAATTC CTAGGTTTCCGCGGTCTCG	cloninig for pEXG2-Δs3661
F_BHI_Del_s02 23	CAGGTCGACTCTAGA GGATCC GAGATCAAGCCGATCCCG	cloninig for pEXG2-Δs0223
R_Del_s0223_f us	CGGTGGAACTCAGCGCGCCGCCCAGGCATTG	cloninig for pEXG2-Δs0223
F_Del_s0223_f us	CTGGGCGGCGCGCTGAGTTCCACCGACATGTCGATG	cloninig for pEXG2-Δs0223
R_ERI_Del_s02 23	GAAATTAATTAAGGTACC GAATTC GCGGATTTCTGGAACGTC	cloninig for pEXG2-Δs0223
F_XbaI_tfoR	CAACTCTCTACTGTT TCTAGA T AGTTGAAAGGACATCCCTC	cloning for pKH24-rpoS^{Vc}::lacZ/TfoR
R_sphI_tfoR	catccgccaaaacagcc gcatgc GTGAGTGATGGTAATAGAG	cloning for pKH24-rpoS^{Vc}::lacZ/TfoR

F_XbaI_Vcr090	CAACTCTCTACTGTT TCTAGA T AGATACACTGCTTCACGA	cloning for pKH24-rpoS^{Vc}::lacZ/Vcr090
R_sphI_Vcr090	catccgccaaaacagcc gcatgc GCACTGAGTCAGGATTTTG	cloning for pKH24-rpoS^{Vc}::lacZ/Vcr090
F_XbaI_Vcr043	CAACTCTCTACTGTT TCTAGA T ACTGTCATCTCGTTAGTC	cloning for pKH24-rpoS^{Vc}::lacZ/Vcr043
R_sphI_Vcr043	catccgccaaaacagcc gcatgc GACAAACCGGTGTTGGTAG	cloning for pKH24-rpoS^{Vc}::lacZ/Vcr043
F_XbaI_svca0830	CAACTCTCTACTGTT TCTAGA T AGGAAGCGGACACGGAAC	cloning for pKH24-rpoS^{Vc}::lacZ/svca0830
R_sphI_svca0830	catccgccaaaacagcc gcatgc CATACGGAAAGATGCCAAG	cloning for pKH24-rpoS^{Vc}::lacZ/svca0830
F_XbaI_vc_ryhB	CAACTCTCTACTGTT TCTAGA T ATCTTAGGGAACAAGTGAAG	cloning for pKH24-rpoS^{Vc}::lacZ/RyhBVc
R_sphI_vc_ryhB	catccgccaaaacagcc gcatgc GAGCGAGAGCGAGAACCA	cloning for pKH24-rpoS^{Vc}::lacZ/RyhBVc
R_rpoS_Ec_a	AAAAAAAAAAAAAAAAAAGCGTACTGGTTGATGTACTGCTG	for enrichment of E. coli rpoS chimeric RNAs
R_rpoS_Ec_b	AAAAAAAAAAAAAAAAAAGGTTTACGGATTTCCCCTTGTAACG	for enrichment of E. coli rpoS chimeric RNAs
R_rpoS_Ec_c	AAAAAAAAAAAAAAAAAACCAACACACGCTGTGTGGCTCC	for enrichment of E. coli rpoS chimeric RNAs
R_rpoS_Ec_d	AAAAAAAAAAAAAAAAAAGACAGATGCTTACTTACTCGCG	for enrichment of E. coli rpoS chimeric RNAs
R_PArpoS-a	AAAAAAAAAAAAAAAAAAGTTGTGACCGTAGGCACTCACG	for enrichment of P. aeruginosa rpoS chimeric RNAs
R_PArpoS-b	AAAAAAAAAAAAAAAAAAGAATCTCGAAGTGCAGCTTCACCC	for enrichment of P. aeruginosa rpoS chimeric RNAs
R_PArpoS-c	AAAAAAAAAAAAAAAAAACAGGAGGAGCACTTCATCATCGTG	for enrichment of P. aeruginosa rpoS chimeric RNAs
R_PArpoS-d	AAAAAAAAAAAAAAAAAACTCGACAGGCCATTCTTCTCCAG	for enrichment of P. aeruginosa rpoS chimeric RNAs
R_rpoS_Vc-a	AAAAAAAAAAAAAAAAAAGTTGCCTTGATCGCCCGCAG	for enrichment of V. cholera rpoS chimeric RNAs
R_rpoS_Vc-b	AAAAAAAAAAAAAAAAAATCCCCCTGGCAACTTTGCGAG	for enrichment of V. cholera rpoS chimeric RNAs
R_rpoS_Vc-c	AAAAAAAAAAAAAAAAAAGCGTTTACGTGCGGCTTCATCAC	for enrichment of V. cholera rpoS chimeric RNAs
R_rpoS_Vc-d	AAAAAAAAAAAAAAAAAAGTCGTATTCGACGTTAAACAGCGC	for enrichment of V. cholera rpoS chimeric RNAs
R_NP_saspA	GTATAGCGTTTTGCTTTGTAAGCCG	Northern blot probe for sAspA
R_NP_asYbiE	AAACAAGGGTAACATAGGATCAATG	Northern blot probe for asYbiE
R_NP_ErsA	GCTTCGTATGGGGAGGGGGAAG	Northern blot probe for ErsA
R_NP_ReaL	GGGCACCGGGTCGCGATCAG	Northern blot probe for ReaL
R_NP_s3661	GCGGGGGCTCGCTGTCCGGGAT	Northern blot probe for s3661
R_NP_s0223	GACGGGGTGATCAGCGCAGC	Northern blot probe for s0223
R_NP_sAdhC	GACGGCTTGTCAGTAATGAATG	Northern blot probe for sAdhC
R_NP_sRmf	GATGGGGCGTGCCTTGGGGAAATC	Northern blot probe for sRmf
R_NP_s0161	AATAGGAGCAATCAAAGTTCGGCG	Northern blot probe for s0161
R_np_tfoR	GACACCTTCGCTTGCTGCCG	northern blot probe for tfoR

R_np_Vcr090	GAGCCAATCTACAATTCATCAGATAG	northern blot probe for Vcr090
R_np_Vcr043	AGTCAGCCTTCAAAAGGAAGTG	northern blot probe for Vcr043
R_np_svca0838	AGTTCTCACTCGCAGCAATCCC	northern blot probe for svca0838
R_np_vcRyhB	ACACTGGAAGCAATGTGAGCAATGT	northern blot probe for RyhBvc
F_speI_IGybiCJ_ctx	GGCCGCTCTAGA ACTAGT GAAGAGACGTTGCAGGC	cloning for pIG-ybiCJ
R_KpnI_IGybiCJ_ctx	CTATAGGGCGAATTG GGTACC CAGTGGATACAGCATCAC	cloning for pIG-ybiCJ
F_araD_Tn5	CGCGCCATGCTTACGCAGATAGTGTTTATCCAGCAG CGTTATTCCGGGGATCCGTCGACC	for deletion of araDABC
R_araC_Tn5	TCATTCACTTTTTCTTCACAACCGGCACGAAACTCGC TCGTGTAGGCTGGAGCTGCTTCG	for deletion of araDABC

Table S4. Strains or plasmids used in this study.

Strain or plasmid	Relevant genotype or description	Source or reference
E. coli		
MG1655	Wild-type	lab stock
MG1655Δ*lacAI*	MG1655 ΔlacAYZI	Thomas Bernhard lab
MG1655Δ*araDC*Δ*lacAI*	MG1655 Δ*lacAYZI*, Δ*araDABC*	This study
SM10	*thi-1 leuB6 supE44 tonA21 lacY1 recA::RP4-2-Tc::Mu Kmr*	lab stock
StellarTM competent cell	*F–, endA1, supE44, thi-1, recA1, relA1, gyrA96, phoA, Φ80d lacZΔ M15, Δ(lacZYA-argF) U169, Δ(mrr-hsdRMS-mcrBC), ΔmcrA, λ–*	Clontech
P. aeruginosa		
PAO1	Wild-type	
PAO1Δ*ersA*	Isogenic deletion strain constructed with pEXG2-Δ*ersA*	This study
PAO1Δ*reaL*	Isogenic deletion strain constructed with pEXG2-Δ*reaL*	This study
PAO1Δ*s3661*	Isogenic deletion strain constructed with pEXG2-Δ*s3661*	This study
PAO1Δ*s0223*	Isogenic deletion strain constructed with pEXG2-Δ*s0223*	This study
PAO1Δ*sr0161*	Isogenic deletion strain constructed with pEXG2-Δ*sr0161*	Zhang *et al* (1)
V. cholera		
C6706 *recA-lacZ**	*recA-,lacZ**	John Mekalanos lab
Plasmids		
pBAD24	Bacterial expression vector, Amp^R	Guzman *et al* (2)
pKH24XS	pBAD24 derived vector for sRNA expression, Amp^R	
pKH24::Z	dual expression vector for sRNA (with PBAD) and lacZ translational fusion protein (with Ptac), Amp^R	
pKH24::6H	dual expression vector for sRNA (with PBAD) and 6His-tag translational fusion protein (with	

	Ptac), Amp^R	
pKH24-rpoS^{Ec}::lacZ	dual expression vector for sRNA (with PBAD) and *rpoS^{Ec}::lacZ* translational fusion protein (with Ptac)	
pKH24-rpoS^{Vc}::lacZ	dual expression vector for sRNA (with PBAD) and *rpoS^{Ec}::lacZ* translational fusion protein (with Ptac)	
pKH24-rpoS^{Vc}::6H	dual expression vector for sRNA (with PBAD) and *rpoS^{Vc}*::*6xHis*-tagged translational fusion protein (with Ptac)	
pPtac-miniCTX::lacZ	*lacZ* transcriptional fusion; *attB* integration construction plasmid, Ptac with two lac operator sites, Tet^R	Han *et al* (3)
pPtac-miniCTX::6H	*6xHis*-tagged transcriptional fusion; attB integration construction plasmid, Ptac with two lac operator sites, Tet^R	Han *et al* (3)
pPtac-miniCTXrpoS^{Pa}::lacZ	*rpoS^{Pa}::lacZ* transcriptional fusion; *attB* integration construction plasmid, Ptac with two lac operator sites, Tet^R	
pPtac-miniCTXrpoS^{Pa}::6H	*rpoS^{Pa}::6xHis*-tagged transcriptional fusion; attB integration construction plasmid, Ptac with two lac operator sites, Tet^R	
pKH24-rpoS^{Ec}::lacZ/asYbiE	dual expression vector for asYbiE (with PBAD) and *rpoS^{Ec}::lacZ* translational fusion protein (with Ptac)	
pKH24-rpoS^{Ec}::lacZ/sAspA	dual expression vector for sAspA (with PBAD) and *rpoS^{Ec}::lacZ* translational fusion protein (with Ptac)	
pKH24-rpoS^{Ec}::lacZ/DsrA	dual expression vector for DsrA (with PBAD) and *rpoS^{Ec}::lacZ* translational fusion protein (with Ptac)	
pKH24-rpoS^{Ec}::lacZ/MgrR	dual expression vector for MgrR (with PBAD) and *rpoS^{Ec}::lacZ* translational fusion protein (with Ptac)	
pKH24-rpoS^{Vc}::lacZ/TfoR	dual expression vector for TfoR (with PBAD) and *rpoS^{Ec}::lacZ* translational fusion protein (with Ptac)	
pKH24-rpoS^{Vc}::lacZ/Vcr090	dual expression vector for Vcr090 (with PBAD) and *rpoS^{Vc}::lacZ* translational fusion protein (with Ptac)	
pKH24-rpoS^{Vc}::lacZ/Vcr043	dual expression vector for Vcr043 (with PBAD) and *rpoS^{Vc}::lacZ* translational fusion protein (with Ptac)	
pKH24-rpoS^{Vc}::lacZ/sVca0830	dual expression vector for sVca0830 (with PBAD) and *poS^{Vc}::lacZ* translational fusion protein (with Ptac)	

pKH24-rpoS^{Vc}::lacZ/RyhB^{Vc}	dual expression vector for RyhB^{Vc} (with PBAD) and *rpoS^{Vc}::lacZ* translational fusion protein (with Ptac)	
pKH24-rpoS^{Vc}::6H/TfoR	dual expression vector for TfoR (with PBAD) and *rpoS^{Vc}::6xHis*-tagged translational fusion protein (with Ptac)	
pKH24-rpoS^{Vc}::6H/Vcr090	dual expression vector for Vcr090 (with PBAD) and *rpoS^{Vc}::6xHis*-tagged translational fusion protein (with Ptac)	
pKH24-rpoS^{Vc}::6H/Vcr043	dual expression vector for Vcr043 (with PBAD) and *rpoS^{Vc}::6xHis*-tagged translational fusion protein (with Ptac)	
pKH24-rpoS^{Vc}::6H/sVca0830	dual expression vector for sVca0830 (with PBAD) and *rpoS^{Vc}::6xHis*-tagged translational fusion protein (with Ptac)	
pKH24-rpoS^{Vc}::6H /RyhB^{Vc}	dual expression vector for RyhB^{Vc} (with PBAD) and *rpoS^{Vc}::6xHis*-tagged translational fusion protein (with Ptac)	
pKH6	pJN105-derivated vector: expression of small RNA with TTS +1, Gen^{R}	Han *et al* (3)
pKH6-ErsA	*P. aeruginosa* PAO1 ErsA expression.	
pKH6-ReaL	*P. aeruginosa* PAO1 ReaL expression.	
pKH6-s3661	*P. aeruginosa* PAO1 s3661 expression.	
pKH6-s0223	*P. aeruginosa* PAO1 s0223 expression.	
pKH6-sRmf	*P. aeruginosa* PAO1 sRmf expression.	
pKH6-sAdhC	*P. aeruginosa* PAO1 sAdhC expression.	
pKH6-sr0161	*P. aeruginosa* PAO1 sr0161 expression.	
pEXG2	ColE1 suicide vector; mob sacB GenR	Rietsch *et al* (4)
pEXG2-ΔersA	pEXG2 with flanking regions to introduce an unmarked ersA	
pEXG2-ΔreaL	pEXG2 with flanking regions to introduce an unmarked reaL	
pEXG2-Δs3661	pEXG2 with flanking regions to introduce an unmarked s3661	
pEXG2-Δs0223	pEXG2 with flanking regions to introduce an unmarked s0223	
mini-CTX1	*P. aeruginosa* PAO1 attB integration construction plasmid, TetR	Hoang *et al* (4)
pIG-ybiCJ	pmini-ctx carrying the intergenic region of ybiC and ybiJ	

References

1. Zhang, Y.F., Han, K., Chandler, C.E., Tjaden, B., Ernst, R.K. and Lory, S. (2017) Probing the sRNA regulatory landscape of P. aeruginosa: post-transcriptional control of determinants of pathogenicity and antibiotic susceptibility. *Mol Microbiol*, **106**, 919-937.
2. Guzman, L.M., Belin, D., Carson, M.J. and Beckwith, J. (1995) Tight regulation, modulation, and high-level expression by vectors containing the arabinose PBAD promoter. *J Bacteriol*, **177**, 4121-4130.
3. Han, K., Tjaden, B. and Lory, S. (2016) GRIL-seq provides a method for identifying direct targets of bacterial small regulatory RNA by in vivo proximity ligation. *Nat Microbiol*, **2**, 16239.
4. Rietsch, A., Wolfgang, M.C. and Mekalanos, J.J. (2004) Effect of metabolic imbalance on expression of type III secretion genes in Pseudomonas aeruginosa. *Infect Immun*, **72**, 1383-1390.

Table S5. sRNA sequence cloned into the sRNA expression vector.

sRNA	Sequence
asYbiE	AGGCAAGGCAACTAAGCCTGCATTAATGCCAACTTTTAGCGCACGGCTCTCTCCCAAGAGCCATTTCCCTGGACCGAATACAGGAATCGTGTTCGGTCTCTTTTTATCTGTTAAAAGCCAGAAGCATTTCCTTCG
DsrA	AACACATCAGATTTCCTGGTGTAACGAATTTTTTAAGTGCTTCTTGCTTAAGCAAGTTTCATCCCGACCCCCTCAGGGTCGGGATTTTTTTATTGTGCATTCAACGATTCACTTCA
MgrR	AGATTCGTTATCAGTGCAGGAAAATGCCTGTTAGCGTAAAAGCAAAACACAAATCTATCCATGCAAGCATTCACCGCCGGTTTACTGGCGGTTTTTTTTCGCCGTCATAAAAATCAGGCCC
sAspA	AATCTGATGCACCCGGCTTACAAAGCAAAACGCTATACTGATGAAAGCGAACAGTAATCGTACAGGGTAGTACAAATAAAAAAGGCACGTCAGATGACGTGCCTTTTTTCTTGTGAGCAGTAACTTAAAAATAA
ErsA	ACGAATGGCTTCTTGAGCCCTTCGATGCTCCTTTGCAGTGTTTAGTGTTGGCAGATTCCTGGACCCCGCCCTAGCGGTCCGGACTTGAACCCCGAACTTCCCCCTCCCCATACGAAGCTCGGGGTTTTTTTGCCTGGAATTCAGGCTTCCTCGAACGCTTCTTG
ReaL	ATCCAGCGCTGTACTATCCCTTCCAGCGCTGATCGCGACCCGGTGCCCGCACCACCGTGCCGCACGAGCCCCGGAGATCCGCACCTCCGGGGCTCACCTTTTGCTCCTCTCGCACCCTCCTTCCCCGGCTGAGCCCCTCCCCCTGCAAATCCGCGACGAATG
s3661	AAGCGCACCACGACGATTCATCCCGGACAGCGAGCCCCCGCACGCTGTTCTCCCCCTGAGCCTCGCCTACCCGGCGGGGCTTTTTTTGTCCTGCGCCAAACAGCC
s0223	ACTTCATCCTCCGCCGGGGCCTGCCGACCACCATCAAGGCCGGCCTGGGCCTCTCCGGCCTGGAGGTGGGGGCGCCGCGCCTTCCGGTGCAGGCGCTGGATACCGAGGGCTGTCGGTATCTGCAGGGGTTGTTGGAAGAGCTGCGCTGATCACCCCGTCAGCGCTTTGACCCGACGGGCCCGGACGACTTGGGGCCCGTCGTTTTTTTTGCCGACGCGCAGCGCGCTCAG
sRmf	AACACGGATAGCACCGATTTCCCCAAGGCACGCCCCATCCGGGCGGCGGGCGCAAGCCCAAGGGCTCCGCAAGGAGCCCTTTTCAATTCCGCCGCGGCAATGCGGCGATGGCGTC
sAdhC	AAGGCGCAGAAGGGCGAGATCCCGCTGGATACCTTCATCACCCACACCATGGGGCTGGAGGACATCAACGAGGCCTTCGAGCTGATGCACGAAGGCAAGAGCATCCGCACCGTCATTCATTACTGACAAGCCGTCGCGGCGGGCCCTGCGGCTCGCCACGACCGTTTCACGGAGGTCACGTGAAGGATTCC
sr0161	ACTTCCCGACGCCGAACTTTGATTGCTCCTATTCGACGTCTTCTCAAGGCCGCTGATACCAGCGGCCTTTTTTTTCGGGTTGCGCCCGGCTGGGCGCCCCATGGGAGCGATCGCC
TfoR	AGTTGAAAGGACATCCCTCCTAGAGACAAGAATTCCAGAGGTGGTATGACCATGTCATACCACTCCGGCAGCAAGCGAAGGTGTCGTTGACACCCGTGGTATAGTCCCCCCGGCTATACCACGATATTTTTTTCCTACTGTTTTACCTCTATTACCATCACTCAC
Vcr043	ACTGTCATCTCGTTAGTCATTACGACTGACATTGTTGAGCAAAGATGACA

	CTTCCTTTTGAAGGCTGACTTTACTTTCTCCTAATCAGGAAACTGATTTTT<br>CAATTGGCGTAAGTTAATTGCTTTACCCATTCCGACCACACAGTTCTGTG<br>TGGTTTTTTTTTGCAAAAAATCCTACCAACACCGGTTTGTC
Vcr090	AGATACACTGCTTCACGAATAGACAACCTTTTGTCCTATCTGATGAATTG<br>TAGATTGGCTCATATGTTTTAAGCGAGTTTAAATAGCTCGCTCTTTTTTTG<br>CCTACGATTTGGCAAAATCCTGACTCAGTGC
sVcr0838	AGGAAGCGGACACGGAACAGGAAAGACCCAAGGATTGGTCATCTTCAGG<br>ACGAAGATTTCGATTGTTCAGGATGAATGGTCGGCAAGGAGAGCAAAGG<br>ACGTTAGCTGGACGCTTAATAACTAGGATGGTTATAAACGGAGAGTTAA<br>TGGACAACTTAATGGACTAAGCGAGTTCTGTCAGGATGGCAGAGGAAGG<br>GACACCGCTAGGAAGGCGATGAAACGGATTGTGCTGAAGGACACAGCAG<br>ACTATCAAGGAATAGATGCAGGGAGCACCTATTAGTAGCGGGATTGCTG<br>CGAGTGAGAACTGAACCCCACTAAGCTTAGGCTTAGTGGGGTTTTCTTTT<br>TTAGCTTCGCTTTTGTCTCTTGGCATCTTTCCGTATG
RyhB^{Vc}	GTCTTAGGGAACAAGTGAAGGTTCACCAGGACGTTAAGTCACCCTTTATC<br>GAGTCATCGATATGCGGTAGCGAAACGGCCGAACTTGAGCAGGTTCTTTT<br>TGACACGACATTGCTCACATTGCTTCCAGTGTAATTTTTAGCTTTTTGGTA<br>AAGCGAATCAAAAAATTGGCTTTGACCTCGTTTGCTAGGCGACATCTTCG<br>GGTGTCGCTTTTTCTTTTTATGTTATGGTTCTCGCTCTCGCTCATGAAGTT<br>ATCC
