# Supplementary material for: Toward a Comprehensive Analysis of Posttranscriptional Regulatory Networks: a New Tool for the Identification of Small RNA Regulators of Specific mRNAs
Source: mBio. 2021 Feb 23;12(1):e03608-20. doi: 10.1128/mBio.03608-20 (PMC8545128; doi:10.1128/mBio.03608-20)
Supplement: FIG S2 [file mbio.03608-20-sf002.pdf]

Fig. S2

A

aspA →

-35 -10 +1

...AAGTCGTTCTGGAACGCGGTCTG**TTGACT**GAAGCGGAACTTGACGA**TATTTT**CTCCGT**<u>A</u>CAGAATC**

TGATGCACCCGGCTTACAAAGCAAAACGCTATACTGATGAAAGCGAACAG<u>taa</u>TCGTACAGGGTAGT

+113

ACAAATAAAAAAGGCACGTCAGATGACGTGCCTTTTT<u>T</u>CTTGTGAGCAGTAACTTAAAAATAACAATCT

AATATCAACTTGTTAAAAAACAAGGAAGGCTAAT**<u>atg...</u>** *dcuA* →

B

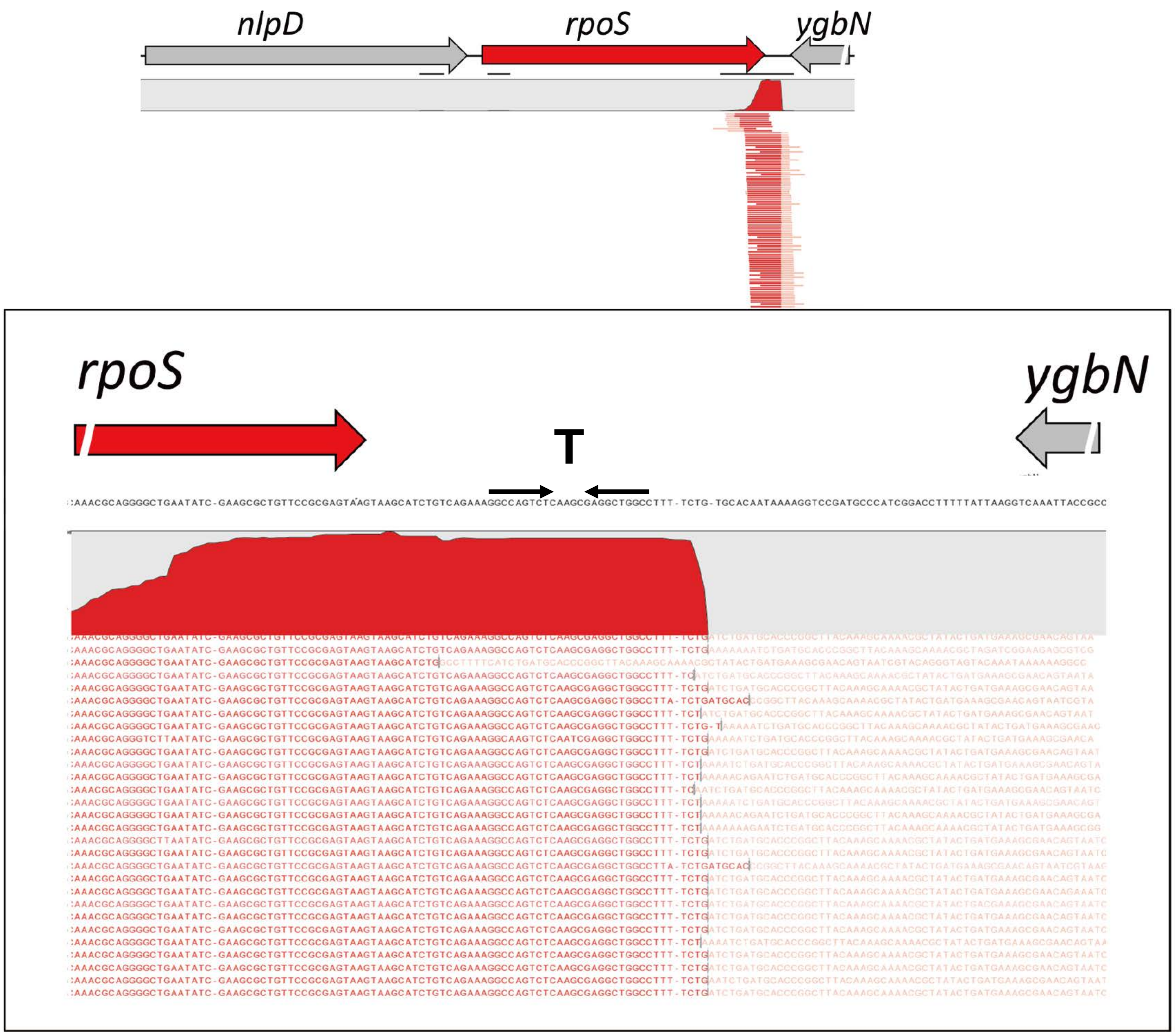

C

```
              +994                +1008
                |                    |
5'-GGU..AGUAA       AUC         AAAGG..CUG-3'  rpoS^Ec
             GUAAGC      UG  UCAG
             ||||||      ||  ||||
             CAUUCG      AC  AGUC
3'-UUU..CGAAA       GCCC   GU      UAAGA-5'      sAspA
                |                    |
               +23                  +6
```
