# Supplementary figures and images for: Toward a Comprehensive Analysis of Posttranscriptional Regulatory Networks: a New Tool for the Identification of Small RNA Regulators of Specific mRNAs
Source: mBio. 2021 Feb 23;12(1):e03608-20. doi: 10.1128/mBio.03608-20 (PMC8545128; doi:10.1128/mBio.03608-20)

Fig. S3

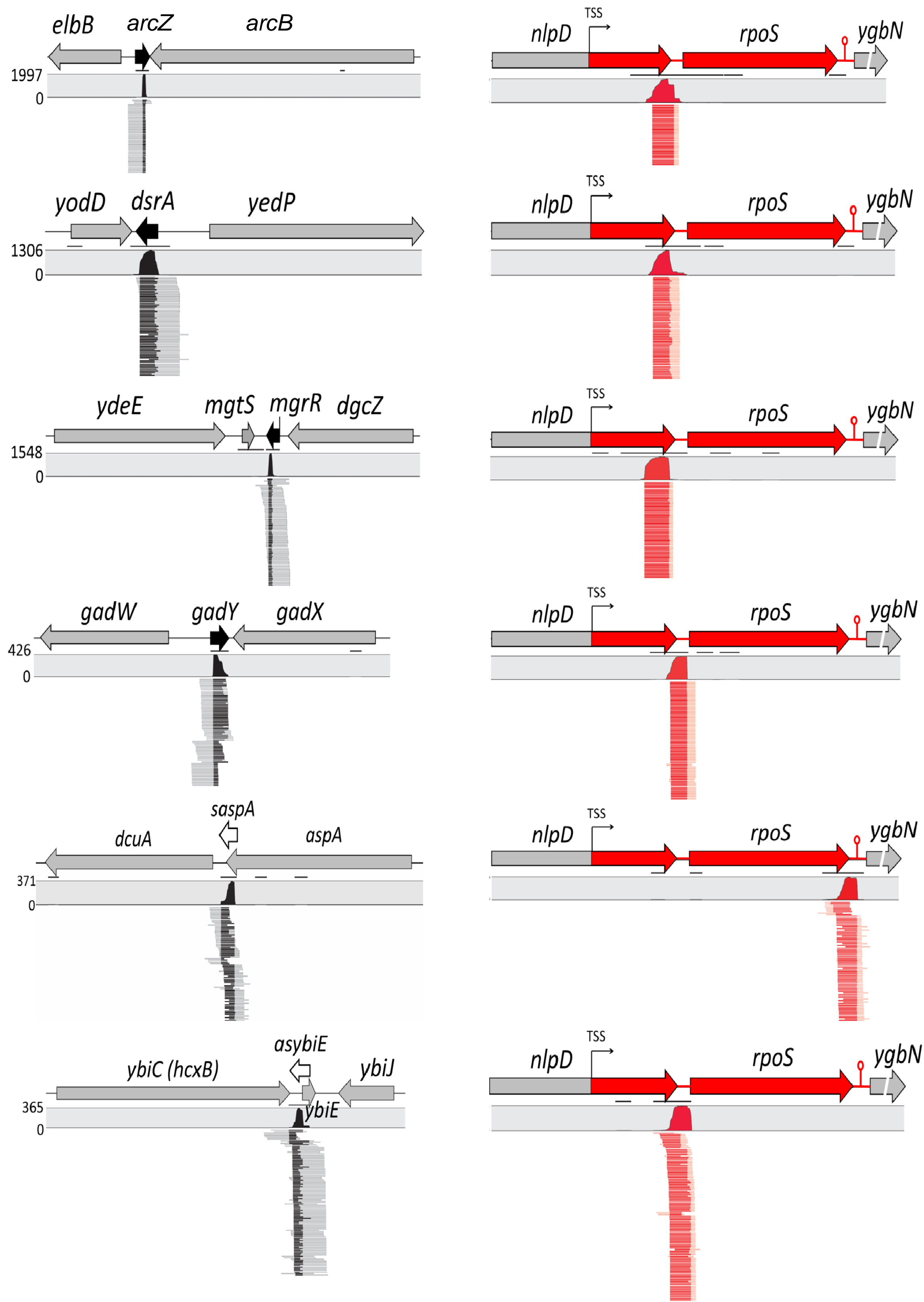

Supplement: FIG S3 [file mbio.03608-20-sf003.pdf]

Fig. S4

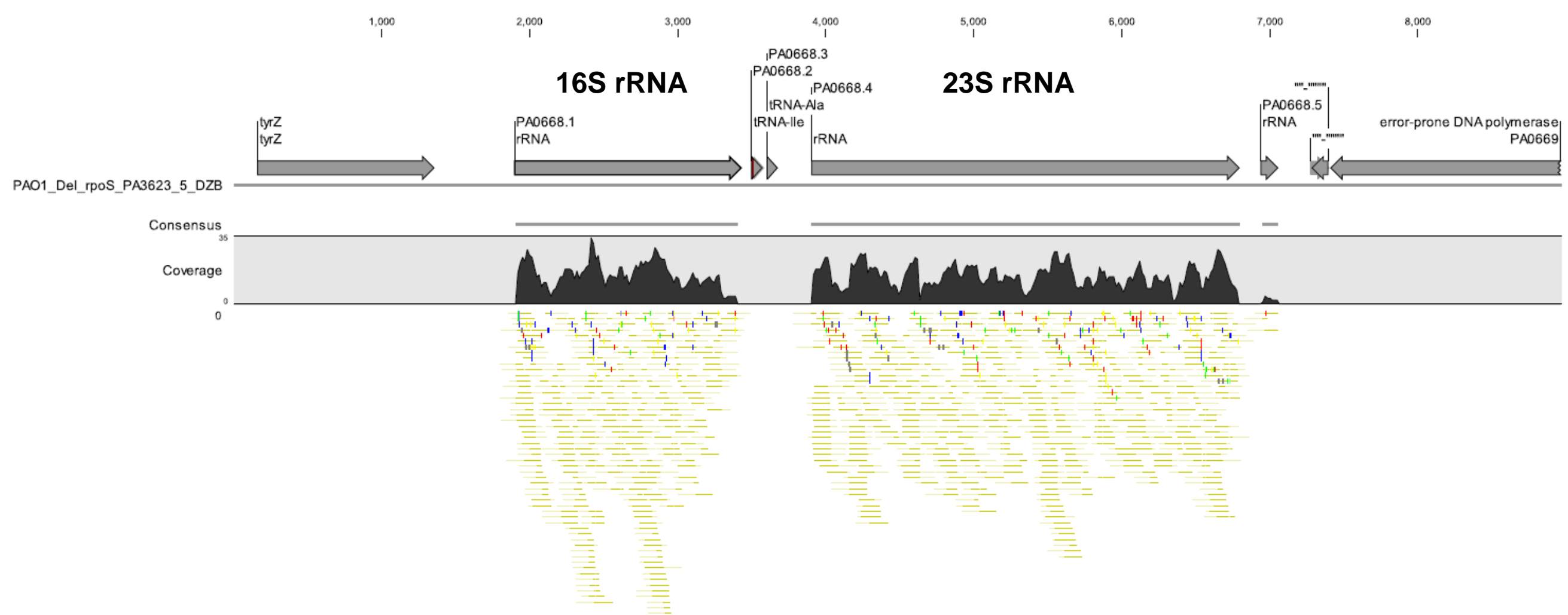

Supplement: FIG S4 [file mbio.03608-20-sf004.pdf]

Fig. S5

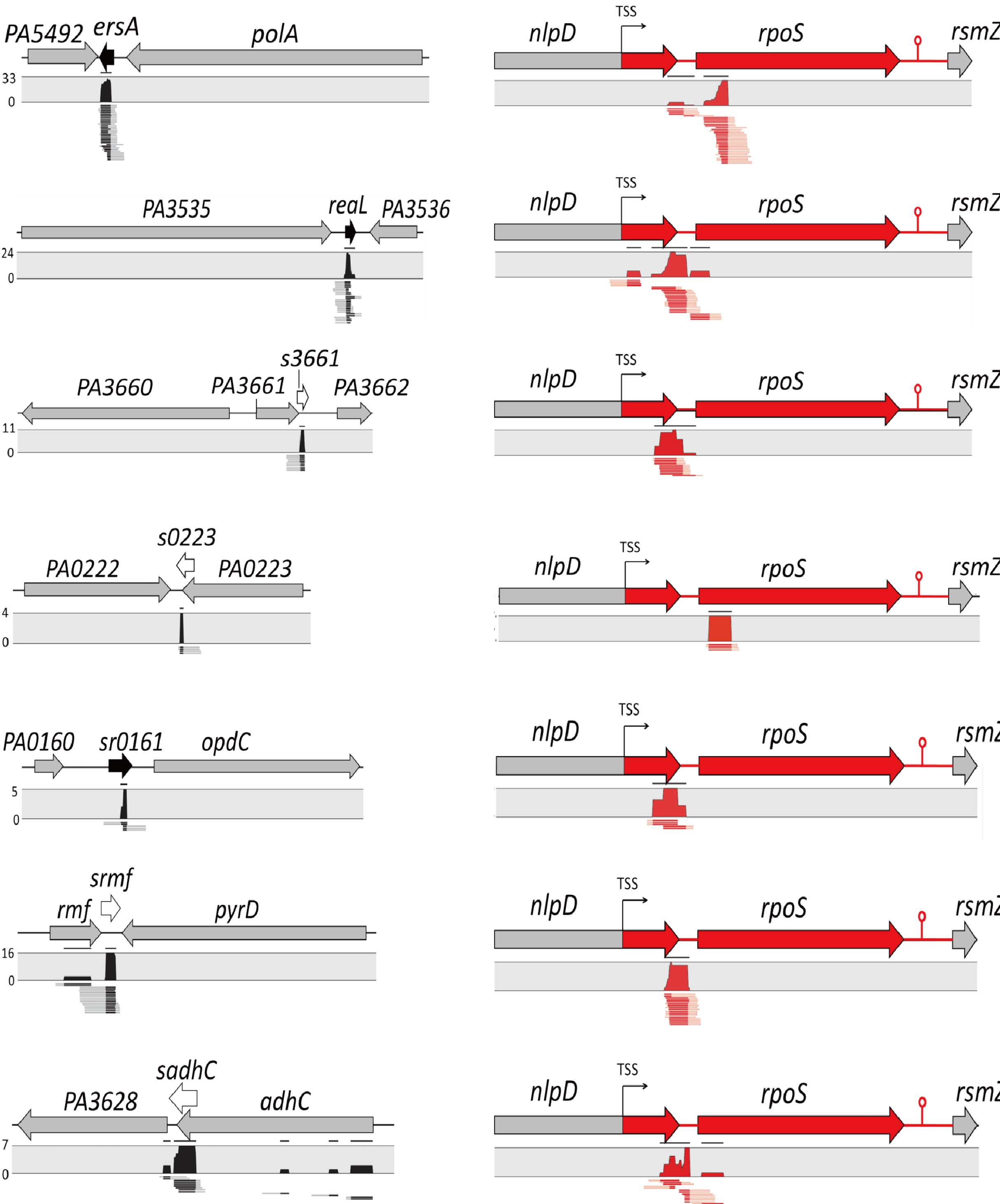

Supplement: FIG S5 [file mbio.03608-20-sf005.pdf]

Fig. S6

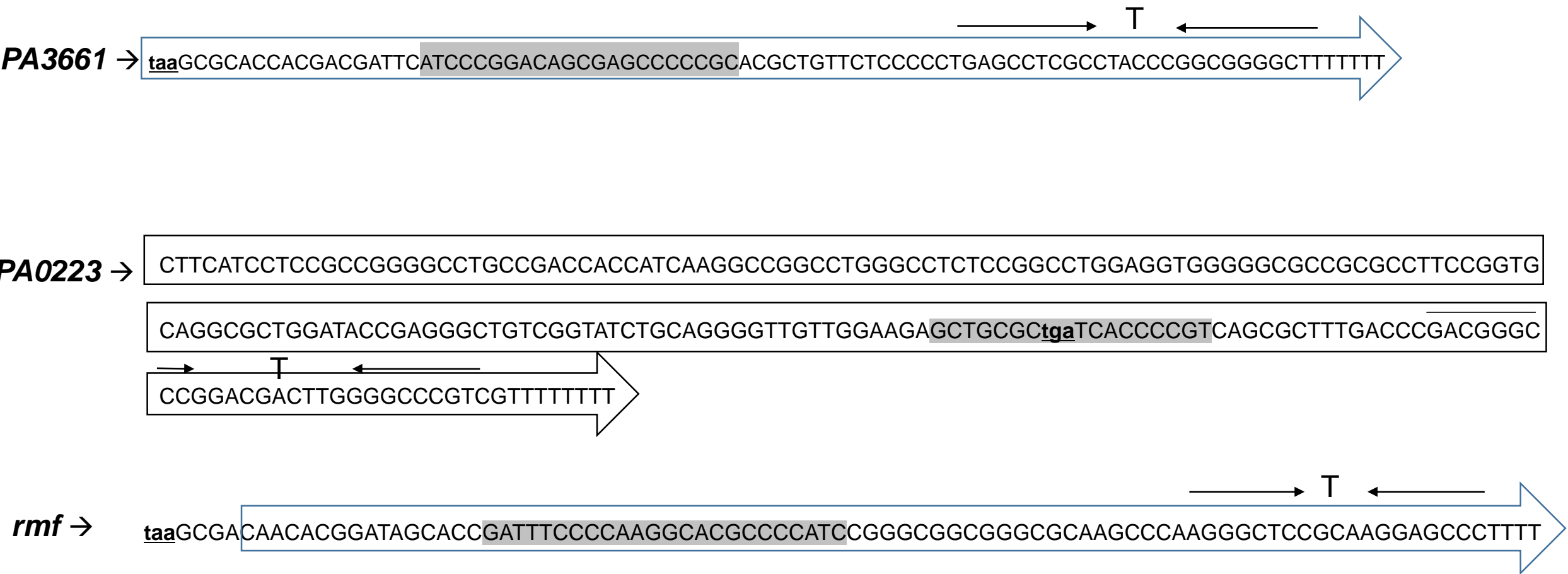

Supplement: FIG S6 [file mbio.03608-20-sf006.pdf]

Fig. S7

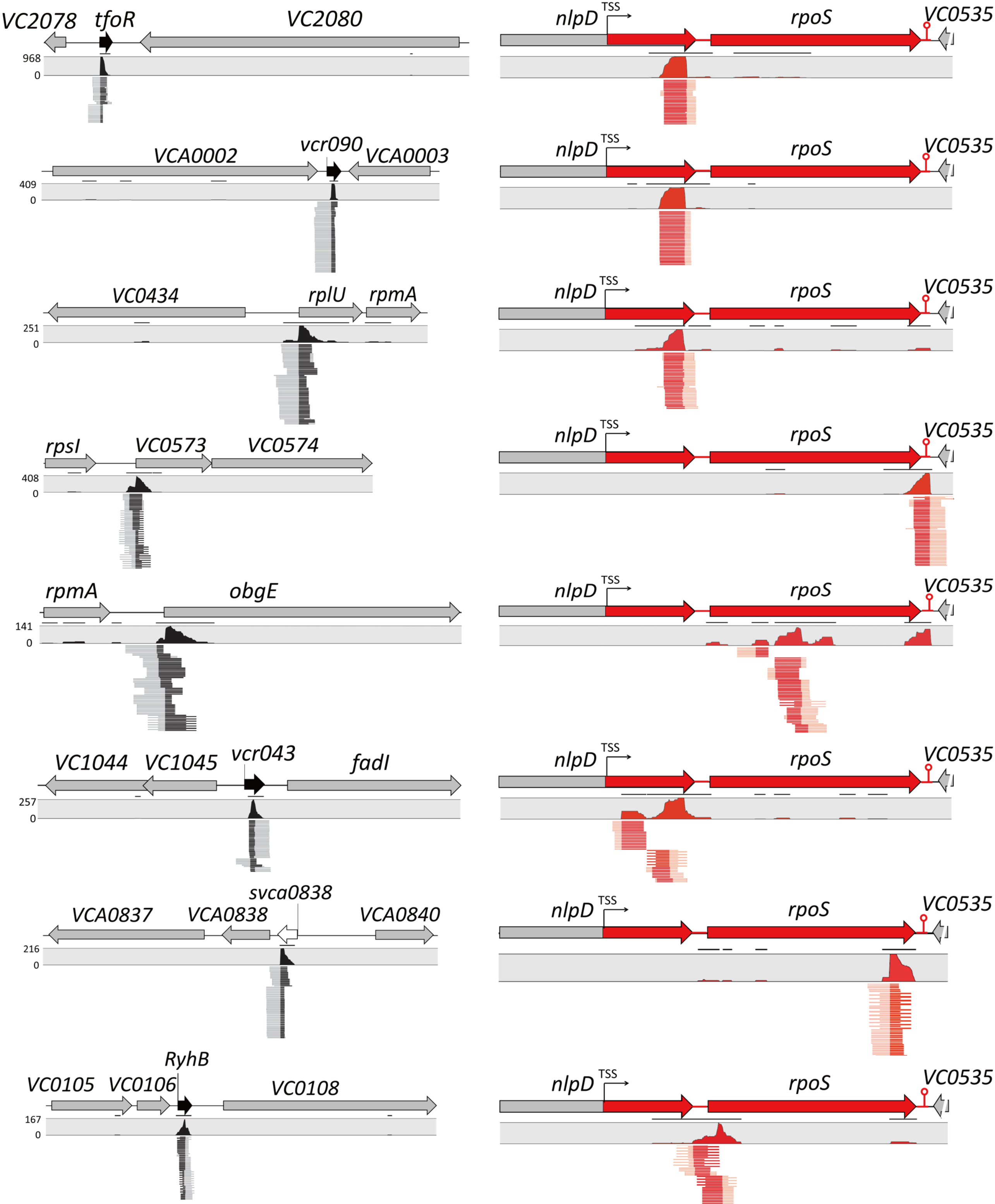

Supplement: FIG S7 [file mbio.03608-20-sf007.pdf]
