# Supplementary material for: Toward a Comprehensive Analysis of Posttranscriptional Regulatory Networks: a New Tool for the Identification of Small RNA Regulators of Specific mRNAs
Source: mBio. 2021 Feb 23;12(1):e03608-20. doi: 10.1128/mBio.03608-20 (PMC8545128; doi:10.1128/mBio.03608-20)
Supplement: TABLE S2 [file mbio.03608-20-st002.pdf]

Table S2.

A. Oligonucleotides used in this study.

Name	Sequence (5' to 3')	Used for
R_pKH24Xblvec	tctagaaacagtagagagttgc	cloning for pKH24XS
F_KH24SphIvec	aagcttggctgttttggc	cloning for pKH24XS
F_DelNsil lacI	cagcttatcatcgatgcaCTCTTCTGCTCCCGAAC	PCR for IacIq and lacZ (or 6His)
R_NsiI_lacZ_6H	caggcacattatgcataattaaccctcactaaagggaac	PCR for IacIq and lacZ (or 6His)
F_ERI_ecrpoS+80aa	CAATATAATGTGTG GAATTC TTCGGGTGAACAGAGTGC	cloning for pKH24-rpoS^{Ec}::lacZ
R_Hd3_ecrpoS+80aa	tgtaaaacgacggcAAGCTT CGCAAAATAAACTTCTTCTTC	cloning for pKH24-rpoS^{Ec}::lacZ
F_ERI_vcrpoS+80aa	CAATATAATGTGTG GAATTC GTCGGTAAACAAAATGTTAATG	cloning for pKH24-rpoS^{Vc}::lacZ (and -::6H)
R_Hd3_vcrpoS+80aa	tgtaaaacgacggcAAGCTT ATAAAGCACTTCTTCTTCGGC	cloning for pKH24-rpoS^{Vc}::lacZ
R_Hnd3_6H_vcrpoS	GTGGTGgccAAGCTT GTTGTCGTATTCGACGTTAAAC	cloning for pKH24-rpoS^{Vc}::6H
F_XbaI_asYbiE+1	CAACTCTCTACTGTT TCTAGA T ATTTCCCTGTCTGTTTGCCG	cloning for pKH24-rpoS^{Ec}::lacZ/asYbiE
R_sphI_pkh24_asYbiE	catccgccaaaacagcc gcatgc GCTTTCTGTCAGCAGTTAGCC	cloning for pKH24-rpoS^{Ec}::lacZ/asYbiE
F_XbaI_sAspA+1	CAACTCTCTACTGTT TCTAGA T AATCTGATGCACCCGGCTTA	cloning for pKH24-rpoS^{Ec}::lacZ/sAspA
R_sphI_pkh24_sAspA	catccgccaaaacagcc gcatgc GTTATTTTTAAGTTACTGCTC	cloning for pKH24-rpoS^{Ec}::lacZ/sAspA
F_XbaI_DsrA+1	CAACTCTCTACTGTT TCTAGA T AACACATCAGATTTCCTG	cloning for pKH24-rpoS^{Ec}::lacZ/DsrA
R_sphI_pkh24_DsrA	catccgccaaaacagcc gcatgc GGATATTCATGACTTCAG	cloning for pKH24-rpoS^{Ec}::lacZ/DsrA
F_XbaI_MgrR+1	CAACTCTCTACTGTT TCTAGA T aGATTCGTTATCAGTGC	cloning for pKH24-rpoS^{Ec}::lacZ/MgrR
R_sphI_pkh24_MgrR	catccgccaaaacagcc gcatgc GGGCCTGATTTTTATGAC	cloning for pKH24-rpoS^{Ec}::lacZ/MgrR
F_ERI_pa_rpoS+80aa	CAATATAATGTGTG GAATTC GCTGCGTCTGGTGGGAC	cloning for pPtac-miniCTX-rpoSPa::lacZ
R_Hd3_pa_rpoS+80aa	tgtaaaacgacggcAAGCTT CTCTTCGGGCGTCAACAGG	cloning for pPtac-miniCTX-rpoSPa::lacZ
R_Hd3_rpoS_ctxH6	GTGGTGgccAAGCTT CTGGAACAGCGCGTCACTCG	cloning for pPtac-miniCTX-rpoSPa::6H
F_XbI_pKH6ErsA+1	CAACTCTCTACTGTT TCTAGA T ACGAATGGCTTCTTGAGCC	cloning for pKH6-ErsA
R_Hnd3_pKH6ErsA+163	CCGCCAAAACAGCC AAGCTT CAAGAAGCGTTCGAGGAAG	cloning for pKH6-ErsA
F_xbaI_pkh6ReaL+1	CAACTCTCTACTGTT TCTAGA T ATCCAGCGCTGTACTATCC	cloning for pKH6-ReaL
R_Hnd3_pkh6R	CCGCCAAAACAGCC AAGCTT	cloning for pKH6-ReaL

eaL	CATTCGTCGCGGATTTGCAG	
F_XbI_pKH6s3661+1	CAACTCTCTACTGTT TCTAGA T AAGCGCACCACGACGATTC	cloning for pKH6-s3661
R_Hnd3_pKH6s3661+104	CCGCCAAAACAGCC AAGCTT GGCTGTTTGGCGCAGGAC	cloning for pKH6-s3661
F_XbaI_pKH6s0223+1	CAACTCTCTACTGTT TCTAGA T ACTTCATCCTCCGCCG	cloning for pKH6-s0223
R_Hnd3_pKH6s0223+229	CCGCCAAAACAGCC AAGCTT CTGAGCGCGCTGCGCGTC	cloning for pKH6-s0223
F_XbI_pKH6-sRmf+1	CAACTCTCTACTGTT TCTAGA T AACACGGATAGCACCGATTTC	cloning for pKH6-sRmf
R_Hnd3_pKH6-sRmf+115	CCGCCAAAACAGCC AAGCTT GACGCCATCGCCGCATTG	cloning for pKH6-sRmf
F_XbaI_pKH6sadhc+1	CAACTCTCTACTGTT TCTAGA T AAGGCGCAGAAGGGCGAG	cloning for pKH6-sadhC
R_Hnd3_pKH6sadhc+140	CCGCCAAAACAGCC AAGCTT GGAATCCTTCACGTGAC	cloning for pKH6-sadhC
F_pKH6_XbaIsr0161	CAACTCTCTACTGTT TCTAGA T ACTTCCCGACGCCGAACTTTG	cloning for pKH6-s0161
R_pKH6_Hnd3sr0161	CCGCCAAAACAGCC AAGCTT GGCGATCGCTCCCATGG	cloning for pKH6-s0161
F_BHI_Del_ErsA	CAGGTCGACTCTAGA GGATCC GGAGGCCCAGGCCTACATC	cloninig for pEXG2-ΔesA
R_Del_ErsA_fus	GTTCGGGGTTCAAGTGAAAAACTTTTCCACGGCACCCTC	cloninig for pEXG2-ΔesA
F_Del_ErsA_fus	GTGCCGTGGAAAAGTTTTTCACTTGAACCCCGAACTTCCC	cloninig for pEXG2-ΔesA
R_ERI_Del_ErsA	GGAAATTAATTAAGGTACCGAATTC AGGGCCTGGAAGTCGC	cloninig for pEXG2-ΔesA
F_BHI_Del_ReaL	CAGGTCGACTCTAGA GGATCC AGCTCGACGCACAGAGCAG	cloninig for pEXG2-ΔreaL
R_Del_ReaL-fus	CTGCAAATATGAGAAAAAGACCTAACTGCTCCTCTCGCACCCTCC	cloninig for pEXG2-ΔreaL
F_Del_ReaL-fus	GGAGGGTGCGAGAGGAGCAGTTAGGTCTTTTTCTCATATTTGCAG	cloninig for pEXG2-ΔreaL
R_ERI_Del_ReaL	GAAATTAATTAAGGTACC GAATTC GGTTTCGGACCAGGGAGGTC	cloninig for pEXG2-ΔreaL
F_BHI_Del_s3661	CAGGTCGACTCTAGA GGATCC CATTCGCACAATCCGTACG	cloninig for pEXG2-Δs3661
R_Del_s3661-fus	CAGGGGGAGAACAGCGTGTTAGCCGCAGGGCTGCTGC	cloninig for pEXG2-Δs3661
F_Del_s3661-fus	CCGCAGCAGCCCTGCGGCTAACACGCTGTTCTCCCCCTG	cloninig for pEXG2-Δs3661
R_ERI_Del_s3661	GAAATTAATTAAGGTACC GAATTC CTAGGTTTCCGCGGTCTCG	cloninig for pEXG2-Δs3661
F_BHI_Del_s0223	CAGGTCGACTCTAGA GGATCC GAGATCAAGCCGATCCCG	cloninig for pEXG2-Δs0223
R_Del_s0223_fus	CGGTGGAACTCAGCGCGCCGCCCAGGCATTG	cloninig for pEXG2-Δs0223
F_Del_s0223_fus	CTGGGCGGCGCGCTGAGTTCCACCGACATGTCGATG	cloninig for pEXG2-Δs0223
R_ERI_Del_s0223	GAAATTAATTAAGGTACC GAATTC GCGGATTTCTGGAACGTC	cloninig for pEXG2-Δs0223
F_XbaI_tfoR	CAACTCTCTACTGTT TCTAGA T	cloning for pKH24-

	AGTTGAAAGGACATCCCTC	rpoS^{Vc}::lacZ/TfoR
R_sphI_tfoR	catccgccaaaacagcc gcatgc GTGAGTGATGGTAATAGAG	cloning for pKH24-rpoS^{Vc}::lacZ/TfoR
F_XbaI_Vcr090	CAACTCTCTACTGTT TCTAGA T AGATACACTGCTTCACGA	cloning for pKH24-rpoS^{Vc}::lacZ/Vcr090
R_sphI_Vcr090	catccgccaaaacagcc gcatgc GCACTGAGTCAGGATTTTG	cloning for pKH24-rpoS^{Vc}::lacZ/Vcr090
F_XbaI_Vcr043	CAACTCTCTACTGTT TCTAGA T ACTGTCATCTCGTTAGTC	cloning for pKH24-rpoS^{Vc}::lacZ/Vcr043
R_sphI_Vcr043	catccgccaaaacagcc gcatgc GACAAACCGGTGTTGGTAG	cloning for pKH24-rpoS^{Vc}::lacZ/Vcr043
F_XbaI_svca0830	CAACTCTCTACTGTT TCTAGA T AGGAAGCGGACACGGAAC	cloning for pKH24-rpoS^{Vc}::lacZ/svca0830
R_sphI_svca0830	catccgccaaaacagcc gcatgc CATACGGAAAGATGCCAAG	cloning for pKH24-rpoS^{Vc}::lacZ/svca0830
F_XbaI_vc_ryhB	CAACTCTCTACTGTT TCTAGA T ATCTTAGGGAACAAGTGAAG	cloning for pKH24-rpoS^{Vc}::lacZ/RyhBVc
R_sphI_vc_ryhB	catccgccaaaacagcc gcatgc GAGCGAGAGCGAGAACCA	cloning for pKH24-rpoS^{Vc}::lacZ/RyhBVc
R_rpoS_Ec_a	AAAAAAAAAAAAAAAAAAGCGTACTGGTTGATGTACTGCTG	for enrichment of E. coli rpoS chimeric RNAs
R_rpoS_Ec_b	AAAAAAAAAAAAAAAAAAGGTTTACGGATTTCCCCTTGTAACG	for enrichment of E. coli rpoS chimeric RNAs
R_rpoS_Ec_c	AAAAAAAAAAAAAAAAAACCAACACACGCTGTGTGGCTCC	for enrichment of E. coli rpoS chimeric RNAs
R_rpoS_Ec_d	AAAAAAAAAAAAAAAAAAGACAGATGCTTACTTACTCGCG	for enrichment of E. coli rpoS chimeric RNAs
R_PArpoS-a	AAAAAAAAAAAAAAAAAAGTTGTGACCGTAGGCACTCACG	for enrichment of P. aeruginosa rpoS chimeric RNAs
R_PArpoS-b	AAAAAAAAAAAAAAAAAAGAATCTCGAAGTGCAGCTTCACCC	for enrichment of P. aeruginosa rpoS chimeric RNAs
R_PArpoS-c	AAAAAAAAAAAAAAAAAACAGGAGGAGCACTTCATCATCGTG	for enrichment of P. aeruginosa rpoS chimeric RNAs
R_PArpoS-d	AAAAAAAAAAAAAAAAAACTCGACAGGCCATTCTTCTCCAG	for enrichment of P. aeruginosa rpoS chimeric RNAs
R_rpoS_Vc-a	AAAAAAAAAAAAAAAAAAGTTGCCTTGATCGCCCGCAG	for enrichment of V. cholera rpoS chimeric RNAs
R_rpoS_Vc-b	AAAAAAAAAAAAAAAAAATCCCCCTGGCAACTTTGCGAG	for enrichment of V. cholera rpoS chimeric RNAs
R_rpoS_Vc-c	AAAAAAAAAAAAAAAAAAGCGTTTACGTGCGGCTTCATCAC	for enrichment of V. cholera rpoS chimeric RNAs
R_rpoS_Vc-d	AAAAAAAAAAAAAAAAAAGTCGTATTCGACGTTAAACAGCGC	for enrichment of V. cholera rpoS chimeric RNAs
R_NP_saspA	GTATAGCGTTTTGCTTTGTAAGCCG	Northern blot probe for sAspA
R_NP_asYbiE	AAACAAGGGTAACATAGGATCAATG	Northern blot probe for asYbiE
R_NP_ErsA	GCTTCGTATGGGGAGGGGGAAG	Northern blot probe for ErsA
R_NP_ReaL	GGGCACCGGGTCGCGATCAG	Northern blot probe for ReaL
R_NP_s3661	GCGGGGGCTCGCTGTCCGGGAT	Northern blot probe for s3661
R_NP_s0223	GACGGGGTGATCAGCGCAGC	Northern blot probe for s0223
R_NP_sAdhC	GACGGCTTGTCAGTAATGAATG	Northern blot probe for sAdhC
R_NP_sRmf	GATGGGGCGTGCCTTGGGGAAATC	Northern blot probe for sRmf

R_NP_s0161	AATAGGAGCAATCAAAGTTCGGCG	Northern blot probe for s0161
R_np_tfoR	GACACCTTCGCTTGCTGCCG	northern blot probe for tfoR
R_np_Vcr090	GAGCCAATCTACAATTCATCAGATAG	northern blot probe for Vcr090
R_np_Vcr043	AGTCAGCCTTCAAAAGGAAGTG	northern blot probe for Vcr043
R_np_svca0838	AGTTCTCACTCGCAGCAATCCC	northern blot probe for svca0838
R_np_vcRyhB	ACACTGGAAGCAATGTGAGCAATGT	northern blot probe for RyhBvc
F_speI_IGybiCJ_ctx	GGCCGCTCTAGA ACTAGT GAAGAGACGTTGCAGGC	cloning for pIG-ybiCJ
R_KpnI_IGybiCJ_ctx	CTATAGGGCGAATTG GGTACC CAGTGGATACAGCATCAC	cloning for pIG-ybiCJ
F_araD_Tn5	CGCGCCATGCTTACGCAGATAGTGTTTATCCAGCAG CGTTATTCCGGGGATCCGTCGACC	for deletion of araDABC
R_araC_Tn5	TCATTCACTTTTTCTTCACAACCGGCACGAAACTCGC TCGTGTAGGCTGGAGCTGCTTCG	for deletion of araDABC

B. Strains or plasmids used in this study.

Strain or plasmid	Relevant genotype or description	Source or reference
E. coli		
MG1655	Wild-type	lab stock
MG1655Δ*lacAI*	MG1655 ΔlacAYZI	Thomas Bernhard lab
MG1655Δ*araDC*Δ*lacAI*	MG1655 Δ*lacAYZI*, Δ*araDABC*	This study
SM10	*thi-1 leuB6 supE44 tonA21 lacY1 recA::RP4-2-Tc::Mu Kmr*	lab stock
StellarTM competent cell	*F–, endA1, supE44, thi-1, recA1, relA1, gyrA96, phoA, Φ80d lacZΔ M15, Δ(lacZYA-argF) U169, Δ(mrr-hsdRMS-mcrBC), ΔmcrA, λ–*	Clontech
P. aeruginosa		
PAO1	Wild-type	
PAO1Δ*ersA*	Isogenic deletion strain constructed with pEXG2-Δ*ersA*	This study
PAO1Δ*reaL*	Isogenic deletion strain constructed with pEXG2-Δ*reaL*	This study
PAO1Δ*s3661*	Isogenic deletion strain constructed with pEXG2-Δ*s3661*	This study
PAO1Δ*s0223*	Isogenic deletion strain constructed with pEXG2-Δ*s0223*	This study
PAO1Δ*sr0161*	Isogenic deletion strain constructed with pEXG2-Δ*sr0161*	Zhang *et al* (1)

V. cholera		
C6706 *recA-lacZ**	*recA-,lacZ**	John Mekalanos lab
Plasmids		
pBAD24	Bacterial expression vector, Amp^R	Guzman *et al* (2)
pKH24XS	pBAD24 derived vector for sRNA expression, Amp^R	
pKH24::Z	dual expression vector for sRNA (with PBAD) and lacZ translational fusion protein (with Ptac), Amp^R	
pKH24::6H	dual expression vector for sRNA (with PBAD) and 6His-tag translational fusion protein (with Ptac), Amp^R	
pKH24-rpoS^{Ec}::lacZ	dual expression vector for sRNA (with PBAD) and *rpoS^{Ec}::lacZ* translational fusion protein (with Ptac)	
pKH24-rpoS^{Vc}::lacZ	dual expression vector for sRNA (with PBAD) and *rpoS^{Ec}::lacZ* translational fusion protein (with Ptac)	
pKH24-rpoS^{Vc}::6H	dual expression vector for sRNA (with PBAD) and *rpoS^{Vc}*::*6xHis*-tagged translational fusion protein (with Ptac)	
pPtac-miniCTX::lacZ	*lacZ* transcriptional fusion; *attB* integration construction plasmid, Ptac with two lac operator sites, Tet^R	Han *et al* (3)
pPtac-miniCTX::6H	*6xHis*-tagged transcriptional fusion; attB integration construction plasmid, Ptac with two lac operator sites, Tet^R	Han *et al* (3)
pPtac-miniCTXrpoS^{Pa}::lacZ	*rpoS^{Pa}::lacZ* transcriptional fusion; *attB* integration construction plasmid, Ptac with two lac operator sites, Tet^R	
pPtac-miniCTXrpoS^{Pa}::6H	*rpoS^{Pa}::6xHis*-tagged transcriptional fusion; attB integration construction plasmid, Ptac with two lac operator sites, Tet^R	
pKH24-rpoS^{Ec}::lacZ/asYbiE	dual expression vector for asYbiE (with PBAD) and *rpoS^{Ec}::lacZ* translational fusion protein (with Ptac)	
pKH24-rpoS^{Ec}::lacZ/sAspA	dual expression vector for sAspA (with PBAD) and *rpoS^{Ec}::lacZ* translational fusion protein (with Ptac)	

pKH24-rpoS^{Ec}::lacZ/DsrA	dual expression vector for DsrA (with PBAD) and *rpoS*^{Ec}*::lacZ* translational fusion protein (with Ptac)	
pKH24-rpoS^{Ec}::lacZ/MgrR	dual expression vector for MgrR (with PBAD) and *rpoS*^{Ec}*::lacZ* translational fusion protein (with Ptac)	
pKH24-rpoS^{Vc}::lacZ/TfoR	dual expression vector for TfoR (with PBAD) and *rpoS*^{Ec}*::lacZ* translational fusion protein (with Ptac)	
pKH24-rpoS^{Vc}::lacZ/Vcr090	dual expression vector for Vcr090 (with PBAD) and *rpoS*^{Vc}*::lacZ* translational fusion protein (with Ptac)	
pKH24-rpoS^{Vc}::lacZ/Vcr043	dual expression vector for Vcr043 (with PBAD) and *rpoS*^{Vc}*::lacZ* translational fusion protein (with Ptac)	
pKH24-rpoS^{Vc}::lacZ/sVca0830	dual expression vector for sVca0830 (with PBAD) and *poS*^{Vc}*::lacZ* translational fusion protein (with Ptac)	
pKH24-rpoS^{Vc}::lacZ/RyhB^{Vc}	dual expression vector for RyhB^{Vc} (with PBAD) and *rpoS*^{Vc}*::lacZ* translational fusion protein (with Ptac)	
pKH24-rpoS^{Vc}::6H/TfoR	dual expression vector for TfoR (with PBAD) and *rpoS*^{Vc}*::6xHis*-tagged translational fusion protein (with Ptac)	
pKH24-rpoS^{Vc}::6H/Vcr090	dual expression vector for Vcr090 (with PBAD) and *rpoS*^{Vc}*::6xHis*-tagged translational fusion protein (with Ptac)	
pKH24-rpoS^{Vc}::6H/Vcr043	dual expression vector for Vcr043 (with PBAD) and *rpoS*^{Vc}*::6xHis*-tagged translational fusion protein (with Ptac)	
pKH24-rpoS^{Vc}::6H/sVca0830	dual expression vector for sVca0830 (with PBAD) and *rpoS*^{Vc}*::6xHis*-tagged translational fusion protein (with Ptac)	
pKH24-rpoS^{Vc}::6H /RyhB^{Vc}	dual expression vector for RyhB^{Vc} (with PBAD) and *rpoS*^{Vc}*::6xHis*-tagged translational fusion protein (with Ptac)	
pKH6	pJN105-derivated vector: expression of small RNA with TTS +1, Gen^{R}	Han *et al* (3)
pKH6-ErsA	*P. aeruginosa* PAO1 ErsA expression.	
pKH6-ReaL	*P. aeruginosa* PAO1 ReaL expression.	
pKH6-s3661	*P. aeruginosa* PAO1 s3661 expression.	
pKH6-s0223	*P. aeruginosa* PAO1 s0223 expression.	
pKH6-sRmf	*P. aeruginosa* PAO1 sRmf expression.	
pKH6-sAdhC	*P. aeruginosa* PAO1 sAdhC expression.	

pKH6-sr0161	*P. aeruginosa* PAO1 sr0161 expression.	
pEXG2	ColE1 suicide vector; mob sacB GenR	Rietsch *et al* (4)
pEXG2-ΔersA	pEXG2 with flanking regions to introduce an unmarked ersA	
pEXG2-ΔreaL	pEXG2 with flanking regions to introduce an unmarked reaL	
pEXG2-Δs3661	pEXG2 with flanking regions to introduce an unmarked s3661	
pEXG2-Δs0223	pEXG2 with flanking regions to introduce an unmarked s0223	
mini-CTX1	*P. aeruginosa* PAO1 attB integration construction plasmid, TetR	Hoang *et al* (4)
pIG-ybiCJ	pmini-ctx carrying the intergenic region of ybiC and ybiJ	

C. sRNA sequence cloned into the sRNA expression vector.

**sRNA**	**Sequence**
asYbiE	AGGCAAGGCAACTAAGCCTGCATTAATGCCAACTTTTAGCGCACGGCTCT CTCCCAAGAGCCATTTCCCTGGACCGAATACAGGAATCGTGTTCGGTCTC TTTTTATCTGTTAAAAGCCAGAAGCATTTCCTTCG
DsrA	AACACATCAGATTTCCTGGTGTAACGAATTTTTTAAGTGCTTCTTGCTTAA GCAAGTTTCATCCCGACCCCCTCAGGGTCGGGATTTTTTTATTGTGCATTC AACGATTCACTTCA
MgrR	AGATTCGTTATCAGTGCAGGAAAATGCCTGTTAGCGTAAAAGCAAAACA CAAATCTATCCATGCAAGCATTCACCGCCGGTTTACTGGCGGTTTTTTTTC GCCGTCATAAAAATCAGGCCC
sAspA	AATCTGATGCACCCGGCTTACAAAGCAAAACGCTATACTGATGAAAGCG AACAGTAATCGTACAGGGTAGTACAAATAAAAAAGGCACGTCAGATGAC GTGCCTTTTTTCTTGTGAGCAGTAACTTAAAAATAA
ErsA	ACGAATGGCTTCTTGAGCCCTTCGATGCTCCTTTGCAGTGTTTAGTGTTGG CAGATTCCTGGACCCCGCCCTAGCGGTCCGGACTTGAACCCCGAACTTCC CCCTCCCCATACGAAGCTCGGGGTTTTTTTTGCCTGGAATTCAGGCTTCCT CGAACGCTTCTTG
ReaL	ATCCAGCGCTGTACTATCCCTTCCAGCGCTGATCGCGACCCGGTGCCCGC ACCACCGTGCCGCACGAGCCCCGGAGATCCGCACCTCCGGGGCTCACCTT TTGCTCCTCTCGCACCCTCCTTCCCCGGCTGAGCCCCTCCCCCTGCAAATC CGCGACGAATG
s3661	AAGCGCACCACGACGATTCATCCCGGACAGCGAGCCCCCGCACGCTGTT CTCCCCCTGAGCCTCGCCTACCCGGCGGGGCTTTTTTTGTCCTGCGCCAA ACAGCC
s0223	ACTTCATCCTCCGCCGGGGCCTGCCGACCACCATCAAGGCCGGCCTGGGC CTCTCCGGCCTGGAGGTGGGGGCGCCGCGCCTTCCGGTGCAGGCGCTGG ATACCGAGGGCTGTCGGTATCTGCAGGGGTTGTTGGAAGAGCTGCGCTG

	ATCACCCCGTCAGCGCTTTGACCCGACGGGCCCGGACGACTTGGGGCCC GTCGTTTTTTTTGCCGACGCGCAGCGCGCTCAG
sRmf	AACACGGATAGCACCGATTTCCCCAAGGCACGCCCCATCCGGGCGGCGG GCGCAAGCCCAAGGGCTCCGCAAGGAGCCCTTTTCAATTCCGCCGCGGC AATGCGGCGATGGCGTC
sAdhC	AAGGCGCAGAAGGGCGAGATCCCGCTGGATACCTTCATCACCCACACCA TGGGGCTGGAGGACATCAACGAGGCCTTCGAGCTGATGCACGAAGGCAA GAGCATCCGCACCGTCATTCATTACTGACAAGCCGTCGCGGCGGGCCCTG CGGCTCGCCACGACCGTTTCACGGAGGTCACGTGAAGGATTCC
sr0161	ACTTCCCGACGCCGAACTTTGATTGCTCCTATTCGACGTCTTCTCAAGGCC GCTGATACCAGCGGCCTTTTTTTTCGGGTTGCGCCCGGCTGGGCGCCCCA TGGGAGCGATCGCC
TfoR	AGTTGAAAGGACATCCCTCCTAGAGACAAGAATTCCAGAGGTGGTATGA CCATGTCATACCACTCCGGCAGCAAGCGAAGGTGTCGTTGACACCCGTG GTATAGTCCCCCCGGCTATACCACGATATTTTTTTCCTACTGTTTTACCTC TATTACCATCACTCAC
Vcr043	ACTGTCATCTCGTTAGTCATTACGACTGACATTGTTGAGCAAAGATGACA CTTCCTTTTGAAGGCTGACTTTACTTTCTCCTAATCAGGAAACTGATTTTT CAATTGGCGTAAGTTAATTGCTTTACCCATTCCGACCACACAGTTCTGTG TGGTTTTTTTTTGCAAAAAATCCTACCAACACCGGTTTGTC
Vcr090	AGATACACTGCTTCACGAATAGACAACCTTTTGTCCTATCTGATGAATTG TAGATTGGCTCATATGTTTTAAGCGAGTTTAAATAGCTCGCTCTTTTTTTG CCTACGATTTGGCAAAATCCTGACTCAGTGC
sVcr0838	AGGAAGCGGACACGGAACAGGAAAGACCCAAGGATTGGTCATCTTCAGG ACGAAGATTTCGATTGTTCAGGATGAATGGTCGGCAAGGAGAGCAAAGG ACGTTAGCTGGACGCTTAATAACTAGGATGGTTATAAACGGAGAGTTAA TGGACAACTTAATGGACTAAGCGAGTTCTGTCAGGATGGCAGAGGAAGG GACACCGCTAGGAAGGCGATGAAACGGATTGTGCTGAAGGACACAGCAG ACTATCAAGGAATAGATGCAGGGAGCACCTATTAGTAGCGGGATTGCTG CGAGTGAGAACTGAACCCCACTAAGCTTAGGCTTAGTGGGGTTTTCTTTT TTAGCTTCGCTTTTGTCTCTTGGCATCTTTCCGTATG
RyhB^{Vc}	GTCTTAGGGAACAAGTGAAGGTTCACCAGGACGTTAAGTCACCCTTTATC GAGTCATCGATATGCGGTAGCGAAACGGCCGAACTTGAGCAGGTTCTTTT TGACACGACATTGCTCACATTGCTTCCAGTGTAATTTTTAGCTTTTTGGTA AAGCGAATCAAAAAATTGGCTTTGACCTCGTTTGCTAGGCGACATCTTCG GGTGTCGCTTTTTCTTTTTATGTTATGGTTCTCGCTCTCGCTCATGAAGTT ATCC

References

1. Zhang, Y.F., Han, K., Chandler, C.E., Tjaden, B., Ernst, R.K. and Lory, S. (2017) Probing the sRNA regulatory landscape of P. aeruginosa: post-transcriptional control of determinants of pathogenicity and antibiotic susceptibility. *Mol Microbiol*, **106**, 919-937.
2. Guzman, L.M., Belin, D., Carson, M.J. and Beckwith, J. (1995) Tight regulation, modulation, and high-level expression by vectors containing the arabinose PBAD promoter. *J Bacteriol*, **177**, 4121-4130.

3. Han, K., Tjaden, B. and Lory, S. (2016) GRIL-seq provides a method for identifying direct targets of bacterial small regulatory RNA by in vivo proximity ligation. *Nat Microbiol*, **2**, 16239.
4. Rietsch, A., Wolfgang, M.C. and Mekalanos, J.J. (2004) Effect of metabolic imbalance on expression of type III secretion genes in Pseudomonas aeruginosa. *Infect Immun*, **72**, 1383-1390.
