# Supplementary material for: Toward a Comprehensive Analysis of Posttranscriptional Regulatory Networks: a New Tool for the Identification of Small RNA Regulators of Specific mRNAs
Source: mBio. 2021 Feb 23;12(1):e03608-20. doi: 10.1128/mBio.03608-20 (PMC8545128; doi:10.1128/mBio.03608-20)
Supplement: TEXT S1 [file mbio.03608-20-s0001.pdf]

Text S1.

Data Analysis of rGRIL-Seq using CLC Genomic Workbench 7.0

1. Import fastq.gz file

1) Select “Import” → ”Illumina”.

2) Select two fastq.gz files (P5 and P7 reads).

3) Select “Paired reads” and save them in a new folder.

2. Trim the adaptor sequences

1) Create the trim adaptor list: “New” → “Trim Adaptor List”→ “Add Row”

2) Select the sequences saved in **Step 1**-3: “Toolbox” → “NGS Core Tools” → “Trim Sequences”.

2) Select the adaptor sequences (P5 and P7 plus index).

3) Create a new folder (i.e. Trimmed) and save them.

3. Map entire rGRIL-Seq reads to *rpoS* gene to identify the reads containing chimeras

In this step, both chimeric and non-chimeric *rpoS* RNA (intact *rpoS*) from the entire reads will be selected.

1) Create a reference genome composed of *rpoS* gene and save it as an *rpoS* reference genome.

- For example, open the *E. coli* MG1655 reference genome (NC_000913) → find *rpoS* sequences including 5’ and 3’ UTR (untranslated region) → Select and save it as *E. coli* rpoS reference genome (Ref1_rpoS_Ec).

- The ends of coordination numbers of *rpoS* reference genomes used in this study are shown below;

  - Ref1_rpoS_Ec (*E. coli*): 2,866,466 to 2,868,118 from MG1655 (NC_000913)
  - Ref1_rpoS_Pa (*P. aeruginosa*): 4,057,863 to 4,059,278 from PAO1 (NC_002516)
  - Ref1_rpoS_Vc (*V. cholerae*): 564,236 to 565,792 from N16961 (NC_002505)

2) Select the reads trimmed and saved in Step **2** and click the right button; “Toolbox” → “NGS Core Tools” → “Map reads to Reference”.

3) Add the sRNA reference genome (i.e., file name: rpoS_Ec)

4) In Mapping options, Set up “Length fraction as 0.16” and “Similarity fraction as 0.98”.

5) In Result handling, check “Create stand-alone read mapping”.

6) Save them.

4. Extract the mapping reads

1) Open the mapping results.

2) Click the right button and choose “Extract Sequences”.

3) Save them as in a new folder (i.e. Extracted).

5. Map the extracted sequences to rpoS-deleted reference genome

In this step, the extracted reads containing chimeric *rpoS* RNA will be mapping to the *rpoS* deleted bacterial reference genome.

1) Create the *rpoS*-deleted reference genome: remove *rpoS* gene from the reference genome and save this modified genome as a new reference genome (i.e., file name: Ref2_DrpoS_Ec). Additional removals of genes are applicable and analyzed it again if an unsuspected mapping is detected after the final mapping of the extracted chimeric reads (Step **4**) to rpoS-deleted reference genome.

- For example, in case of *E. coli rpoS*, the flanking genes (*ygbN*-*rpoS*-*nlpD*) of *rpoS* (ends of coordination number: 2,865,101 to 2,868,892) containing were deleted with *rpoS* from MG1655 (NC_000913) due to the observation of unsuspected mapping of *rpoS* chimeric reads at the flanking genes. In case of *P. aeruginosa rpoS*, additional sequences in two genes (*rpoD*: 634,781 to 635,041 and *mreB*: 5,013,246 to 5,013,262) were deleted due to the observation of the sequence similarity of *rpoS* with these genes along with deletion of flanking genes of *rpoS* (*rsmZ-rpoS-PA3623-pcm-surE*: 4,057,483 to 4,061,362).

- The ends of coordination numbers of the sequences deleted from the genome to create *rpoS*-deleted reference genome in this study are shown below;

  - Ref2_DrpoS_Ec (*E. coli*): 2,865,101 to 2,868,892 from MG1655 (NC_000913)
  - Ref2_DrpoS_Pa (*P. aeruginosa*): 4,057,483 to 4,061,362 / 634,781 to 635,041 / 5,013,246 to 5,013,262 from PAO1 (NC_002516)
  - Ref2_DrpoS_Vc (*V. cholerae*): 563,723 to 568,420 from N16961 (NC_002505)

2) Generate the gene track with this reference genome (Ref2_DrpoS): click the right button “Toolbox” → “Track tools” → “Convert to Track” → ”Select the genome” → “Select ‘Gene’ in Annotation type” → Save them.

3) Map the extract reads generated in Step **4** to the *rpoS*-deleted reference genome as shown in Step **3**-2).

4) Save them in a new folder (i.e. Mapping to DrpoS_Ec).

6. Create statistics for target regions

1) Select the mapped reads generated in Step **5** and click the right button: “Toolbox” → “Resequencing Analysis” → “Create Statistics for target region” → ”Select the gene track as Track of Target Regions” → Save.

2) Open the coverage generated Step **6**-1) and click “Create Track List”.

3) Click the right button on the track and select “Open this track”.

4) The target region coverage track will be shown in table view.

5) Analyze the target genes with coverage of mapping.
